# Supplementary material for: A 0.05 m Change in Inertial Measurement Unit Placement Alters Time and Frequency Domain Metrics during Running
Source: Sensors (Basel). 2024 Jan 19;24(2):656. doi: 10.3390/s24020656 (PMC10820910; doi:10.3390/s24020656)
Supplement: Supplementary file 1 [file sensors-24-00656-s001.zip › sensors-2749179-supplementary.pdf]

Supplemental Materials

for

"A 0.05 m Change in Inertial Measurement Unit Placement Alters Time and Frequency Domain Metrics during Running"

Sections

A. IMU processing details
B. Time series plots
C. Frequency domain analyses
D. Wearable coordinate system results
E. References

A. IMU processing details

A brief overview of our IMU data processing is provided in the main text. Here, we expand on those processing details. After collecting raw data from each IMU, the data were downloaded and processed offline using the following steps:

A.1. Calibration

A.2. Quiet period identification

A.3. Bias removal

A.4. Saturation correction

A.5. Low-pass filtering

A.6. Drift correction

A.7. Orientation estimation and gravity subtraction

A.8. Coordinate system transformation.

A.1. Calibration

All IMU data collected during this experiment were corrected with IMU-specific calibration matrices. These matrices were calculated by conducting a calibration procedure that ensured each IMU accurately expressed accelerations and angular velocities in an orthogonal coordinate system that was oriented square to the IMU housing.

To do so, each IMU was secured to a centrifuge (ClearPath MCVC, Teknic, Victor, NY, USA) with custom 3-D printed jigs (SOLIDWORKS 2019, Dassault Systèmes, Vélizy-Villacoublay, France) and calibrated in 6 orientations at 16 known accelerations (from 0 to 41.42 g where 1 g = 9.8 m/s^2 [1, 2]) and angular velocities (from 0 to 78.54 rad/s). Adapting methods from Coolbaugh et al. [3], known data (K) from the centrifuge and measured data (M) from the IMU were used to calculate 3 × 7 calibration matrices for each IMU (C; three signed magnitude terms, three absolute magnitude terms,

and one bias term per axis) and quantify the sensor accuracy and precision with a hold-back procedure after subtracting out biases observed during a quiet period (B).

$$C * (M + B) = K \quad \text{Equation S1}$$

One potential limitation of this procedure is that it treats each triaxial sensor independently (primary accelerometer, secondary accelerometer, and gyroscope) and assumes their values do not affect each other. This assumption was tested while piloting the calibration procedure by quantifying inter-sensor dependencies between the primary accelerometer and gyroscope and between the secondary accelerometer and gyroscope. Observed dependencies were negligible and independent sensor calibration matrices yielded the best results; thus, we felt confident using this approach (which avoids the indeterminacy of the primary and secondary accelerometer having the same K values).

After calibration, IMU primary accelerometer errors were ≤ 0.01 ± 0.04 g, secondary accelerometer errors were ≤ 0.05 ± 0.07 g, and gyroscope errors were ≤ 0.01 ± 0.01 rad/s.

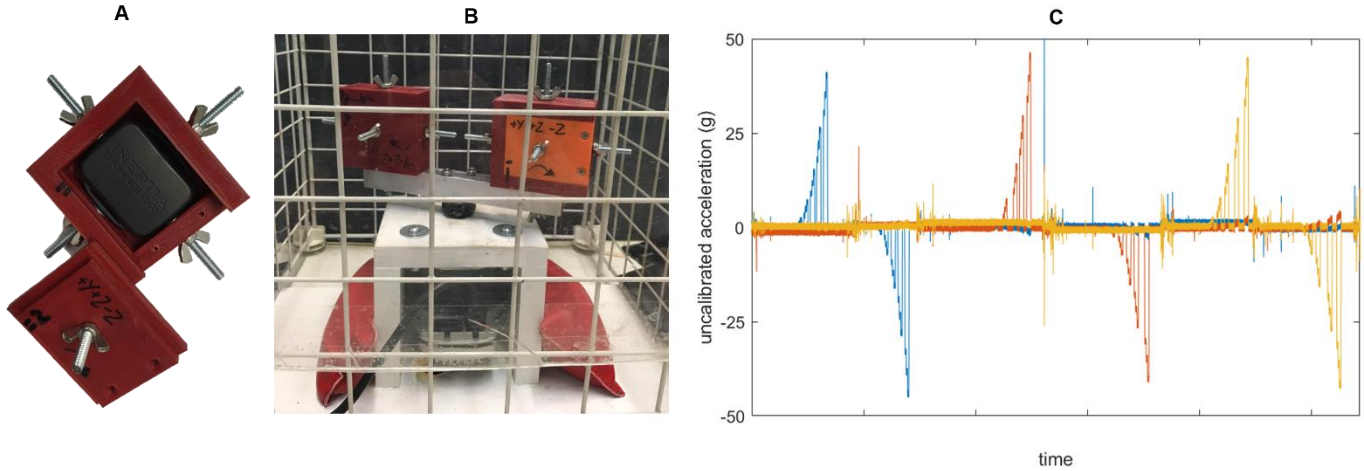

Figure S1: (A) An IMU in 3-D-printed housing. Computer-aided design software was used to ensure IMUs were friction fit square to their housing. **(B)** Two IMUs in their 3-D-printed housings mounted on the centrifuge. IMUs were checked for square with an engineer's square and level with a bullseye level. **(C)** Example of measured triaxial accelerations for the secondary accelerometer (M). The 16 accelerations being applied to the IMU in each of 6 orientations correspond to known (K) values from the centrifuge. Accelerations between each orientation correspond to the IMU being repositioned on the centrifuge and checked for square and level.

A.2. Quiet period identification

Quiet periods were identified throughout data collection (e.g., participant resting, participant preparing at the start of the runway, participant standing while receiving instruction) and used to periodically check for changes in bias (as bias can vary with battery life and temperature) and reset orientation algorithms (as orientation estimates are prone to drift over prolonged periods, discussed further below). These quiet periods were defined as any period where…

$$\omega_{resultant} < 0.5\, rad/s \quad \text{Equation S2}$$

$$j_{resultant} < 0.01\, m/s^3 \quad \text{Equation S3}$$

…for at least 100 ms.

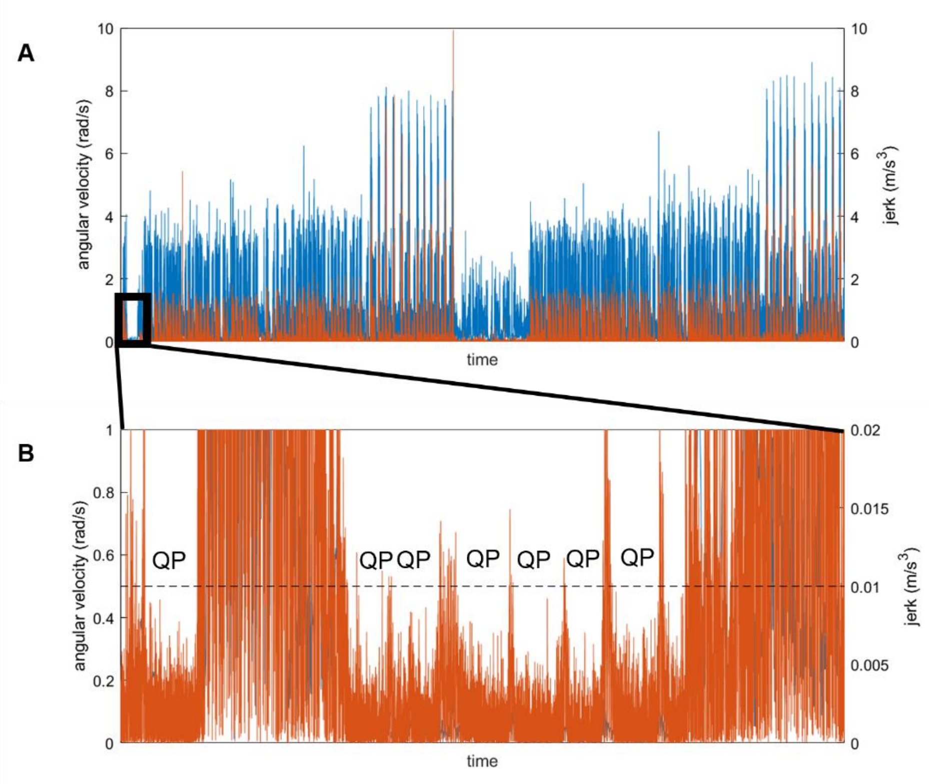

Figure S2: (A) Resultant angular velocity (blue) and jerk (orange) at the sacrum across an entire data collection for a randomly selected example participant. **(B)** Zoomed in to show quiet periods (QP) where resultant angular velocities are < 0.5 rad/s and resultant jerks are < 0.01 m/s^3 for at least 100 ms. Thresholds noted with the dashed horizontal line.

A.3. Bias removal

When the IMU is quiet, we know that it is not accelerating or rotating and thus, the only thing loading the axes should be the gravity vector. Based on this knowledge, we can create a temporary inertial coordinate system based on gravity:

$$Y = \frac{\sum_{first\ quiet\ frame}^{last\ quiet\ frame} a_{calibrated}}{n_{quiet\ frames}} \qquad \text{Equation S4}$$

We can express Y as a unit vector, then make X and Z orthogonal unit vectors (with arbitrary sense). Using these vectors, we can create a temporary rotation matrix that will align our data with gravity:

$$R_{temp} = \begin{bmatrix} X \\ Y \\ Z \end{bmatrix} \qquad \text{Equation S5}$$

We can then express our data in this temporary inertial coordinate system (and given our calibration, we know that the axes of the accelerometer and gyroscope are exactly aligned so the same rotation matrix can be used for both):

$$a_{temp} = R_{temp} * a_{calibrated} \qquad \text{Equation S6}$$

Given the IMU is quiet, in every frame a_{temp} and ω_{temp} should now equal [0 1 0] g and [0 0 0] rad/s, respectively. Thus, we can calculate bias (B) in acceleration and angular velocity as the average deviation from those values across the quiet period:

$$B_a = \frac{\sum_{first\ quiet\ frame}^{last\ quiet\ frame}(a_{temp} - [0\ 1\ 0])}{n_{quiet\ frames}}$$ Equation S7

We can then remove bias and re-express our data in its original coordinates:

$$a_{debias} = a_{calibrated} - R_{temp}^{-1} B_a$$ Equation S8

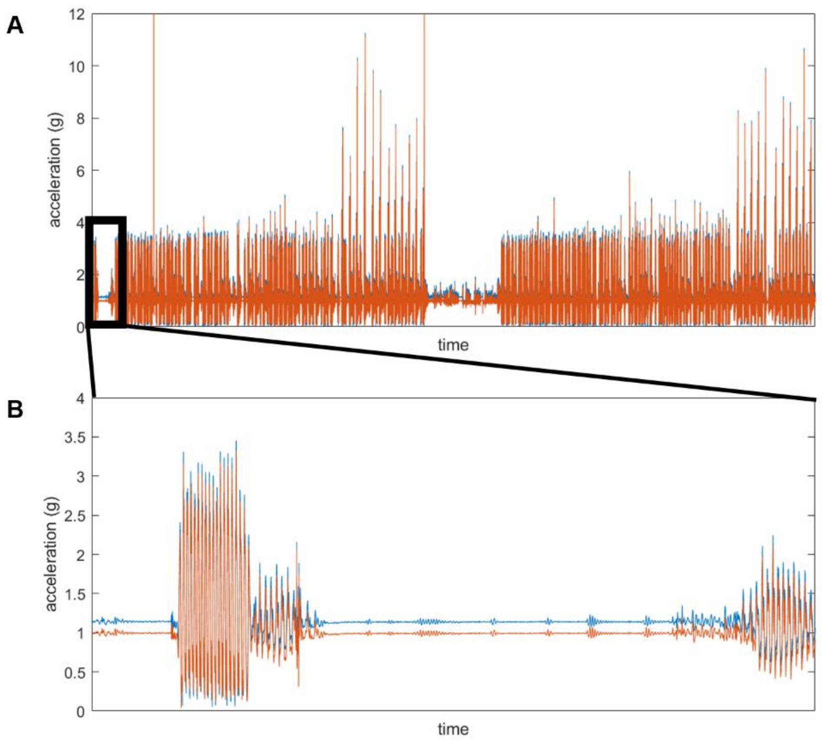

Figure S3: **(A)** Uncorrected resultant sacral acceleration (blue) and de-biased resultant sacral acceleration (orange) across the entire data collection for the randomly selected participant. **(B)** Zoomed in to show that uncorrected resultant acceleration does not equal 1 g during quiet periods, while de-biased acceleration equals exactly 1 g.

A.4. Saturation correction

Our IMU contained two tri-axial accelerometers with different ranges. The primary accelerometer had a range of ± 16 g while the secondary accelerometer had a range of ± 100 g. Although ± 16 g is a large enough range to capture the majority of accelerations at the tibia and sacrum during running, we wanted to ensure that saturation did not occur, particularly at the tibia [4]. Thus, we used a threshold of |a| > 15.5 g and replaced any value above this threshold in our primary accelerometer with the corresponding frame from our secondary accelerometer (these values were highly correlated across the ± 16 g range that they could both measure). The remaining secondary accelerometer data were then discarded.

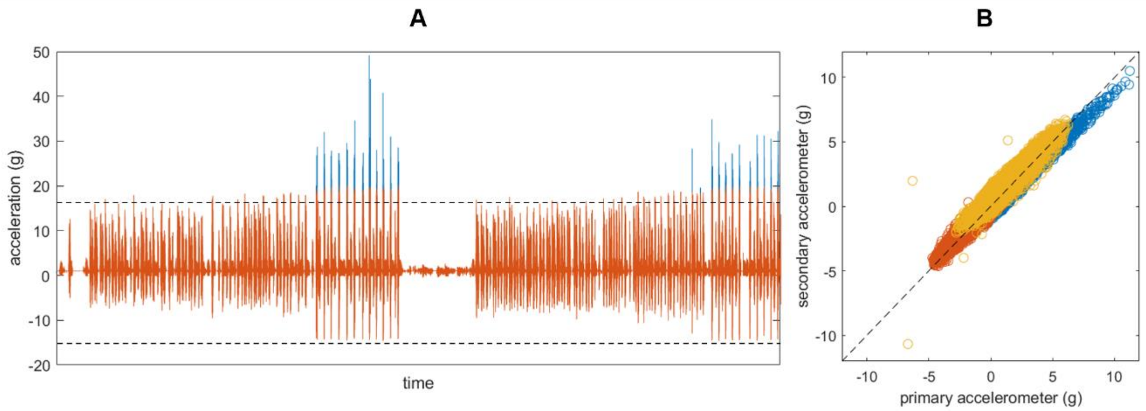

Figure S4. (A) Tibia ~longitudinal axis accelerations from primary accelerometer (orange) and secondary accelerometer (blue) across the entire data collection for the example participant. The horizontal black line indicates data outside the primary accelerometer's range (defined as |a| > 15.5 g). **(B)** Secondary accelerometer measurements plotted against primary accelerometer measurements for each axis (different colors). Black dashed diagonal line indicates perfect agreement. In this randomly selected example data, correlations between each axis ranged from r = 0.82 to 0.96. In general, correlations across the ranges shared between primary and secondary accelerometers were ≥ 0.90.

A.5. Low pass filtering

Next, accelerations and angular velocities were filtered with a 4th-order 50 Hz low-pass Butterworth filter.

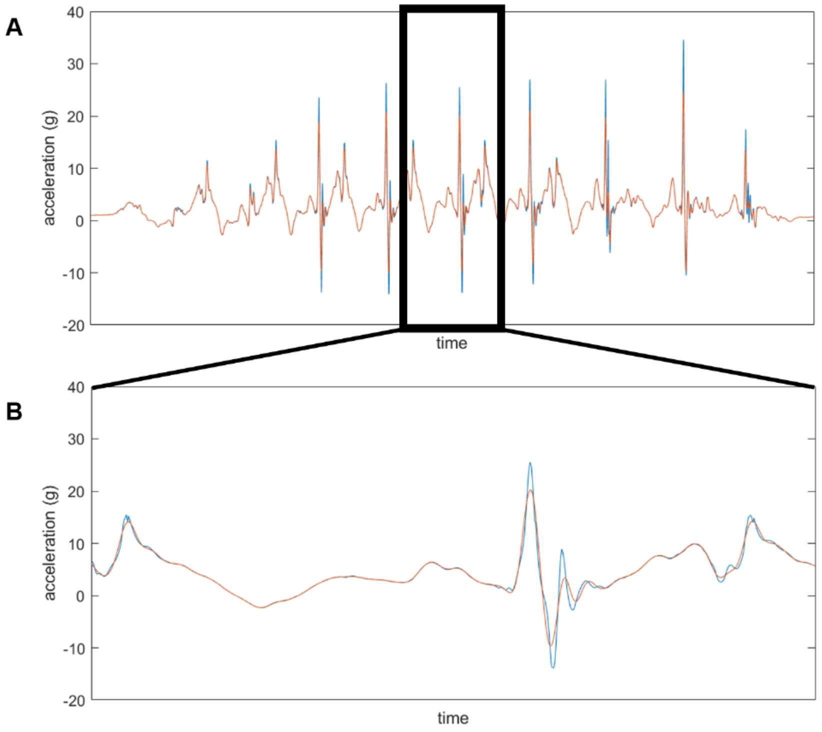

Figure S5: (A) Worst-case example of unfiltered (blue) and filtered (orange) ~longitudinal tibial acceleration (that experienced saturation and is now composed of data from the primary and secondary accelerometers). **(B)** Zoomed in on ~one step to better visualize differences between filtered and unfiltered signals. Filter parameters were chosen to qualitatively balance the preservation of major signal features (particularly peak magnitudes and locations) with the removal of high-frequency noise.

A.6. Drift correction

Angular velocity measured by IMUs is prone to drift. This drift makes it difficult to integrate angular velocities and calculate the orientation of an IMU in space. Several sensor fusion algorithms have been developed to correct this drift including Kalman filters [5], Mahoney filters [6], and Madgwick filters [7]. We explored the use of each of these filters and found that converting our data to quaternion representation and entering it into a Madgwick filter (with beta set to 0.05 and no magnetometer fusion due to the amount of magnetic interference in our lab) was the most successful in eliminating drift in a “worst case” recreation of our experimental conditions (an 80 minute data capture with extreme angular rotations and accelerations and no quiet period corrections yielded a 1.66 rad rotation error accumulated across the entire 80-minute duration). The code we adapted to execute the Madgwick filter is freely available from x-io at:

https://x-io.co.uk/open-source-imu-and-ahrs-algorithms/

And from MATLAB at:

https://www.mathworks.com/products/sensor-fusion-and-tracking.html

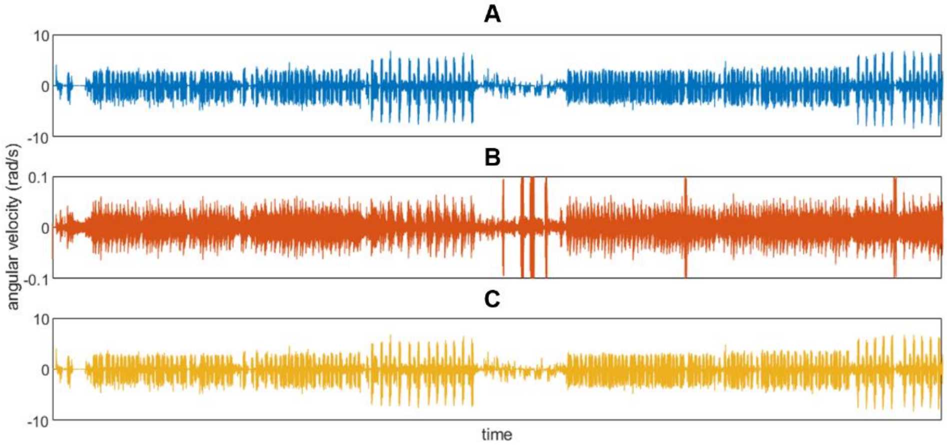

Figure S6: **(A)** Uncorrected angular velocity about the ~longitudinal axis of the sacrum (blue) for the entire data collection of an example participant. **(B)** Difference between uncorrected and Madgwick filter-estimated angular velocities (orange). **(C)** Madgwick filter-estimated angular velocity about the ~longitudinal axis of the sacrum (yellow).

A.7. Orientation estimation and gravity subtraction

After drift-correcting angular velocity with the Madgwick filter, we create a rotation matrix based on the loading of gravity during quiet periods (see A.3 above) then used it to create a “tilt-corrected” coordinate system (see A.8 below). Then, between each quiet period, we used the angular velocity to calculate changes in orientation based on Equations 2 and 3 in McGinnis and Perkins [8]. This provided a rotation matrix from the wearable coordinate system to the “tilt-corrected” coordinate system for each time step.

Using these time-varying rotation matrices, acceleration data for each frame were expressed in the “tilt-corrected” coordinate system, then 1 g was subtracted from the y axis (in line with gravity). This procedure removed the gravity component from the accelerometer data. To create the wearable and segment coordinate systems (in the next step), data were then re-expressed in their original coordinate system using the inverse of the time-varying rotation matrices.

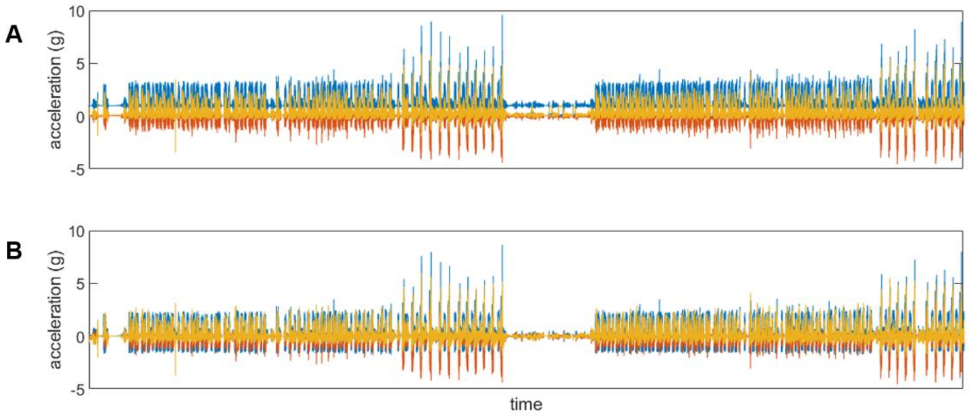

Figure S7. **(A)** Uncorrected acceleration of the sacrum for the entire data collection of a randomly selected example participant (colors represent different axes). **(B)** Acceleration of the sacrum after subtracting 1 g from the y axis in the "tilt-corrected" coordinate system and then re-expressing in the wearable coordinate system (colors represent different axes).

A.8. Coordinate system definition

Finally, data were expressed in three different coordinate systems for analysis. First, data were expressed in the Wearable Coordinate System (WCS). This is not the raw coordinate system of the IMU. Rather, all data were corrected with the calibration matrices described in A.1 above. These calibration matrices ensured that data were expressed in orthogonal axes aligned with the IMU housing. The IMU housing was positioned such that, during quiet standing, the WCS axes were oriented roughly in the direction of progression (+x), the longitudinal axis (+y), and to the right (+z).

Data were also expressed in a Segment Coordinate System (SCS). This coordinate system was defined using an approach described in the Supplemental Material of Cain et al. [9] which can be found at:

http://dx.doi.org/10.1016/j.gaitpost.2015.10. 022

In brief, accelerations during a quiet standing trial were used to define a gravity vector (similar to A.3 above), assuming that the segment was aligned with gravity during the standing trial. This gravity-based vector was defined as the proximal-distal axis (+y proximal). Then, a period of steady-state running was manually selected from the data set. Angular velocities from this period were entered into a Principal Component Analysis and the Principal Component accounting for the most variability in angular velocity was selected to represent the average axis of rotation. During running, the average axis of rotation is assumed to correspond to the medial-lateral axis. We define this as the z axis (+z right). The anterior-posterior axis is then defined as the cross-product of y and z (+x anterior). Finally, the z axis is recalculated as the cross-product of x and y to ensure orthogonality. These three unit vectors were then used to create a rotation matrix that transforms data from the WCS to the SCS. The rotation matrix was then plotted to visualize differences between the WCS and SCS and ensure a solution consistent with our knowledge of the IMU placement had been reached (i.e., axes were oriented correctly).

Finally, data were expressed in a pseudo-global system similar to Cain et al.'s "tilt-corrected" coordinate system (TCCS) [9]. First, we created a rotation matrix based on the loading of gravity during quiet periods (see A.3 above). This rotation matrix expresses data with the y axis aligned with gravity during quiet standing (+y vertical). Next, the acceleration of each axis was double integrated to obtain displacement, then entered into a Principal Component Analysis. The Principal Component

accounting for the most variation in displacement was taken as the projection of the direction of progression onto the horizontal plane (+x direction of progression). Then, the projection of the medial-lateral axis onto the horizontal plane was defined as the cross product of x and z (+z right). The x axis was then recalculated to ensure orthogonality. These three unit vectors were then used to create a rotation matrix and multiplied by the time-varying rotation matrices described in A.7 to express data in the TCCS. Thus, TCCS data are always expressed with y aligned with gravity but with x and z free to rotate about y as the participant moves.

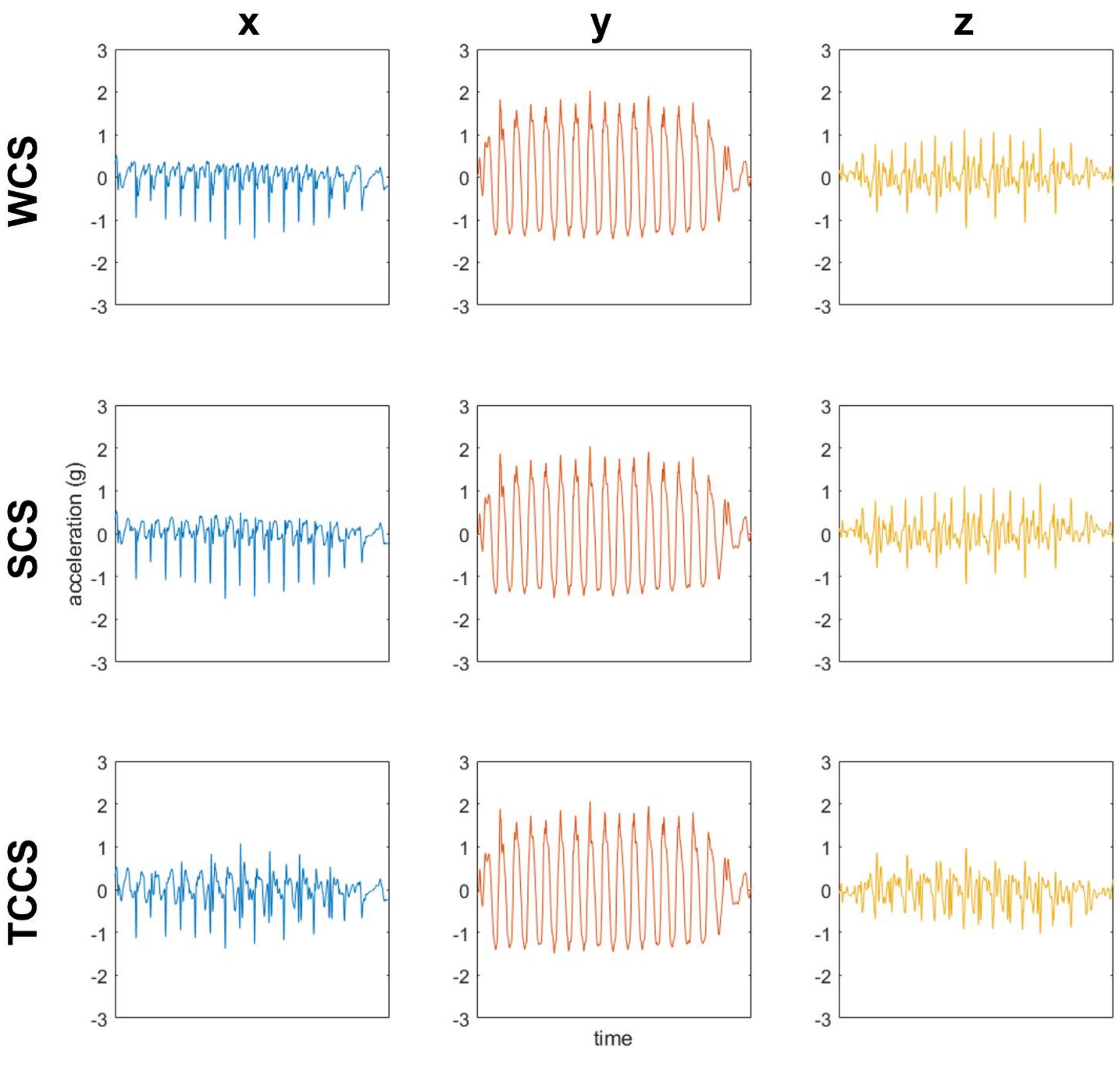

Figure S8: All plots show sacral accelerations from a randomly selected participant. The top row shows data in the Wearable Coordinate System (WCS). The middle row shows data in the Segment Coordinate System. The bottom row shows data in the Tilt-Corrected Coordinate System (TCCS). The first column shows x axis data (blue). The second column shows y axis data (orange). The third column shows z axis data (yellow). Due to the similarities between all three coordinate systems at the sacrum, discrepancies are minor.

<u>B. Time series plots</u>

Means and standard deviations were calculated and plotted for each axis of the reference and misplaced acceleration and angular velocity signals. Data are time-normalized such that the right stance goes from 0-30% of stride and the right swing (right terminal contact to right initial contact) goes from 31-100% of stride (stance was 29.96 ± 5.82% of stride in our data).

B.1. Shank acceleration

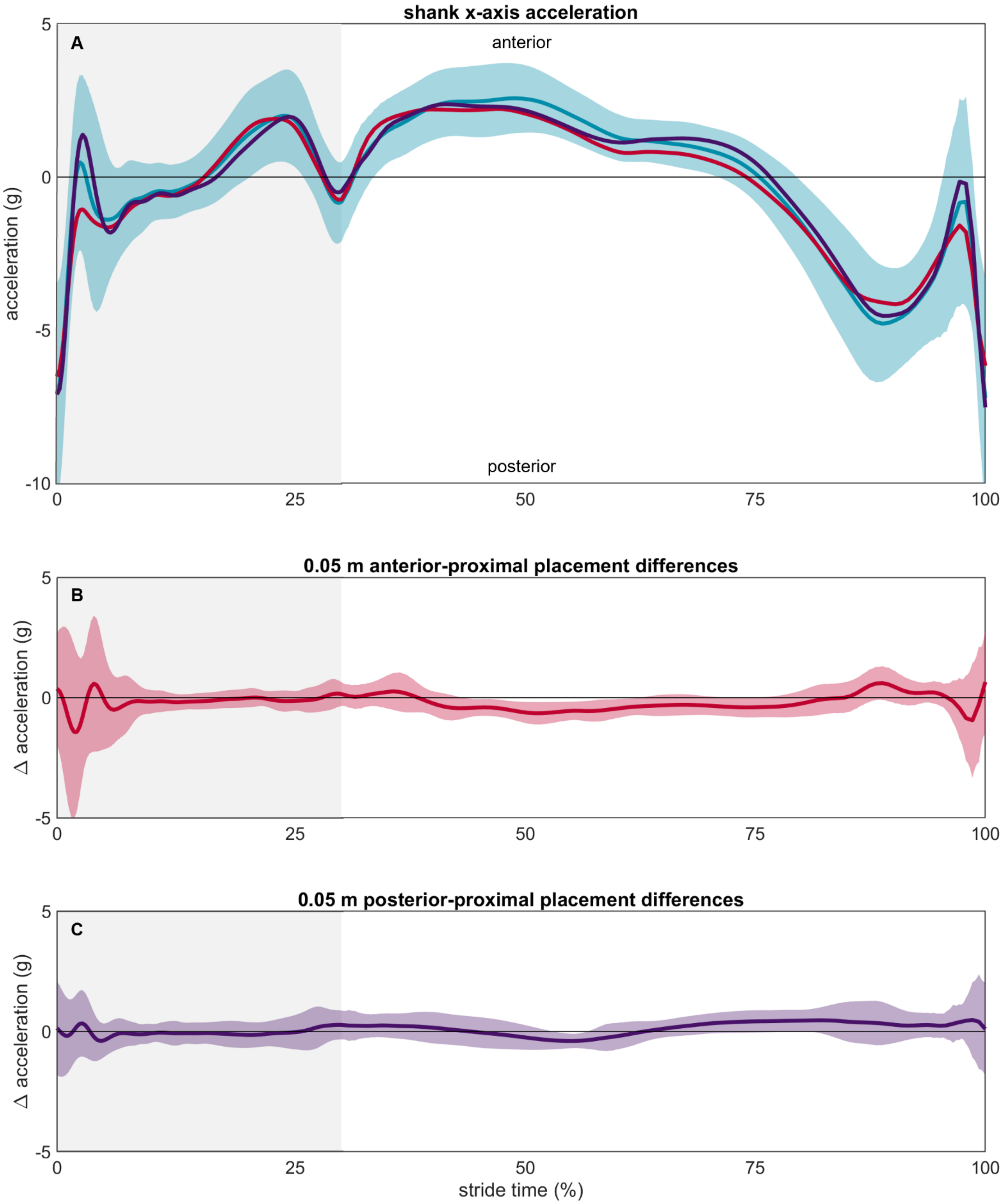

Figure S9: **(A)** Shank x axis accelerations measured at the reference (blue), anterior-proximal misplaced (red), and posterior-proximal (purple) IMUs. The light blue area represents ± 1 SD about the reference mean. The light grey background represents stance while the white background represents swing. Differences between the reference and **(B)** anterior-proximal and **(C)** posterior-proximal IMUs are shown in the panels below.

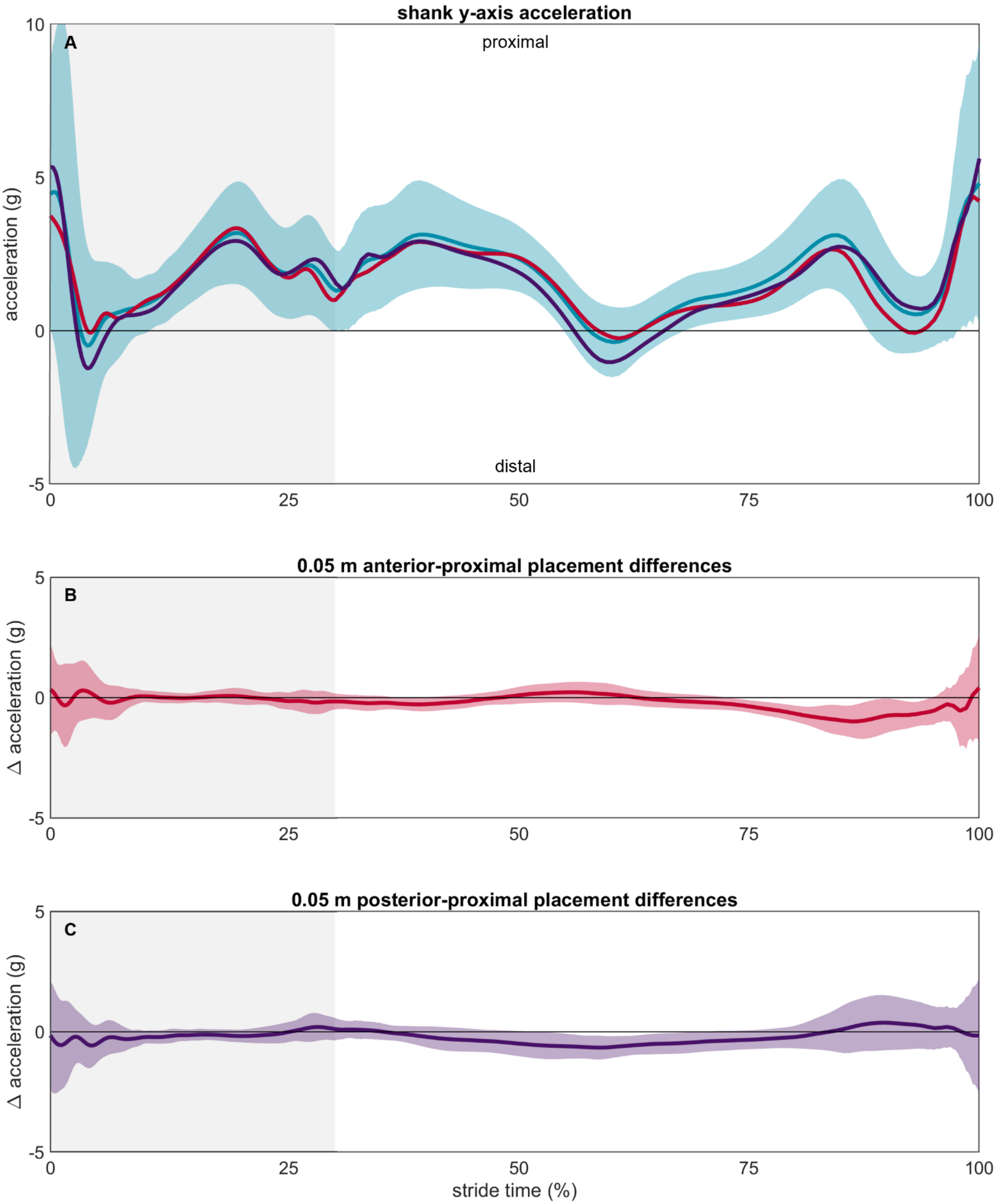

Figure S10: **(A)** Shank y axis accelerations measured at the reference (blue), anterior-proximal misplaced (red), and posterior-proximal (purple) IMUs. The light blue area represents ± 1 SD about the reference mean. The light grey background represents stance while the white background represents swing. Differences between the reference and **(B)** anterior-proximal and **(C)** posterior-proximal IMUs are shown in the panels below.

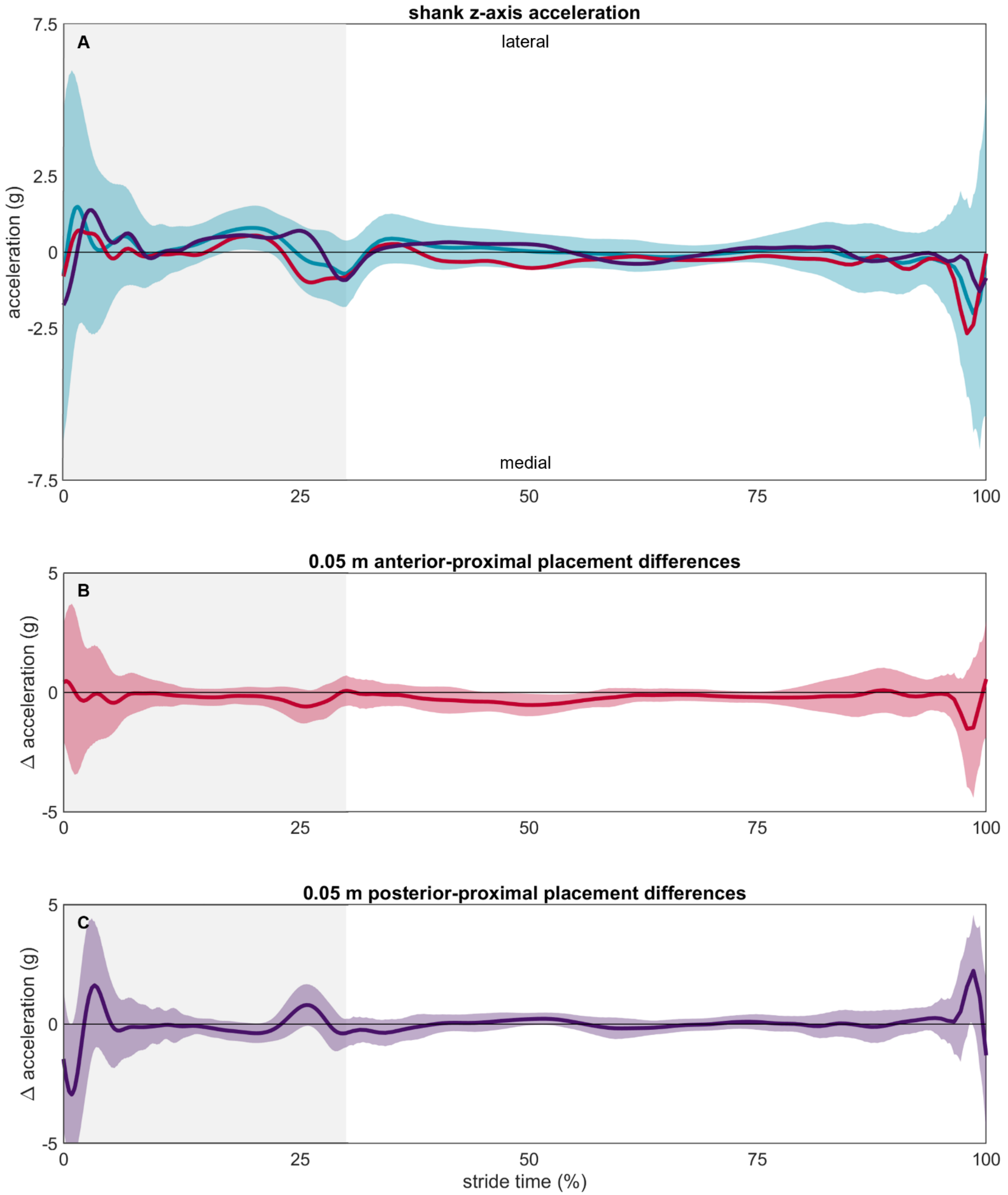

Figure S11: **(A)** Shank z axis accelerations measured at the reference (blue), anterior-proximal misplaced (red), and posterior-proximal (purple) IMUs. The light blue area represents ± 1 SD about the reference mean. The light grey background represents stance while the white background represents swing. Differences between the reference and **(B)** anterior-proximal and **(C)** posterior-proximal IMUs are shown in the panels below.

B.2. Shank angular velocity

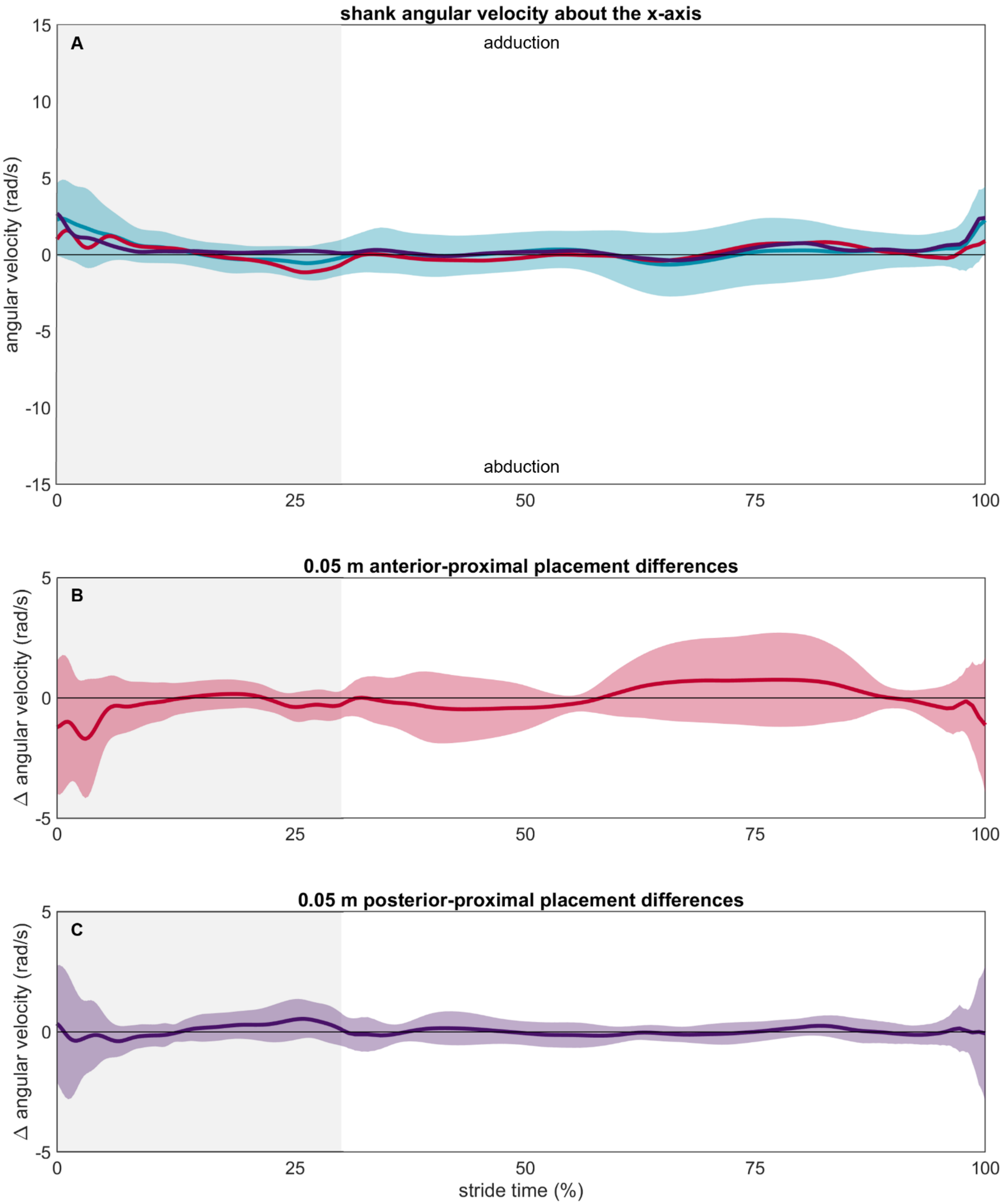

Figure S12: **(A)** Shank x axis angular velocities measured at the reference (blue), anterior-proximal misplaced (red), and posterior-proximal (purple) IMUs. The light blue area represents ± 1 SD about the reference mean. The light grey background represents stance while the white background represents swing. Differences between the reference and **(B)** anterior-proximal and **(C)** posterior-proximal IMUs are shown in the panels below.

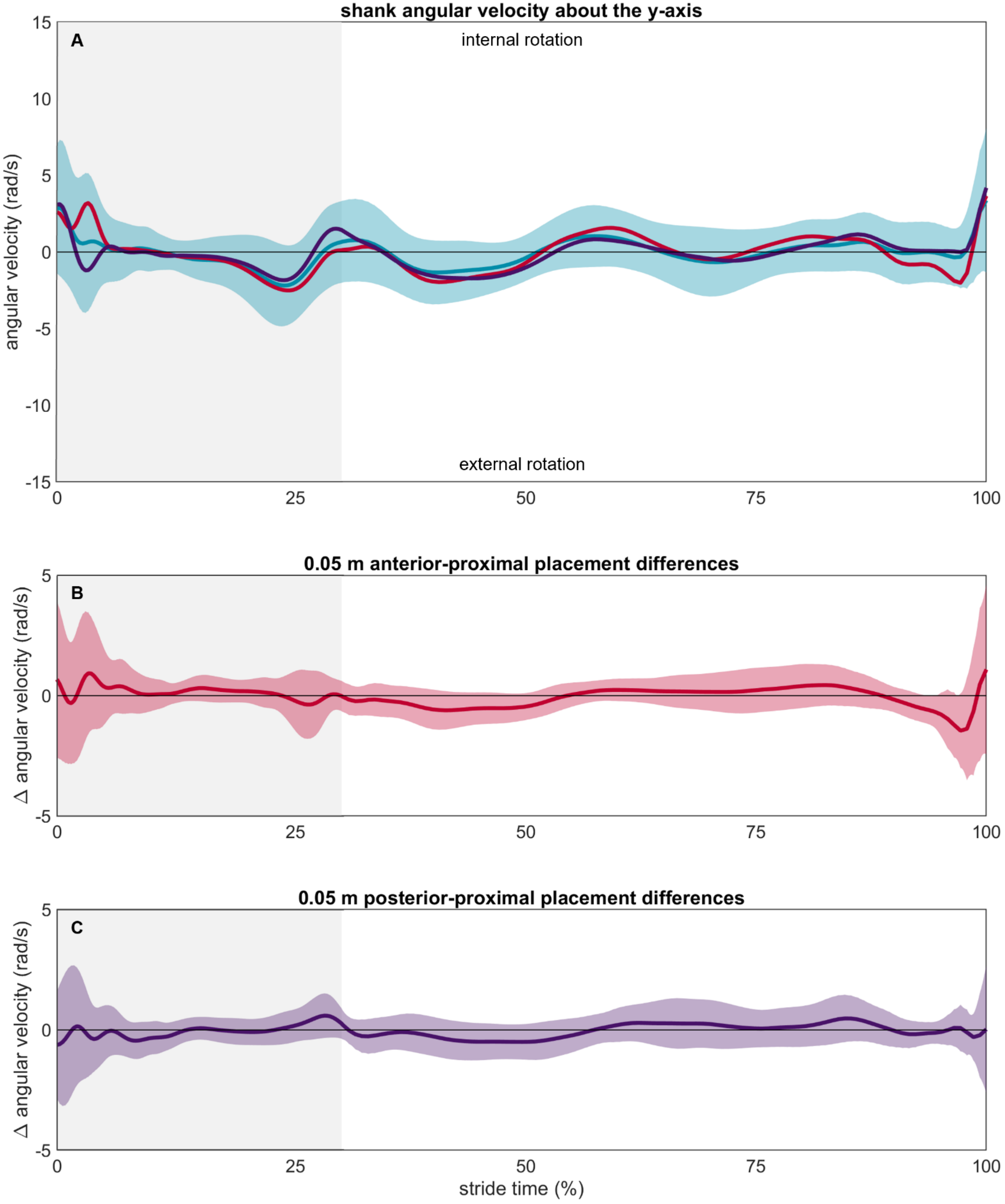

Figure S13: **(A)** Shank y axis angular velocities measured at the reference (blue), anterior-proximal misplaced (red), and posterior-proximal (purple) IMUs. The light blue area represents ± 1 SD about the reference mean. The light grey background represents stance while the white background represents swing. Differences between the reference and **(B)** anterior-proximal and **(C)** posterior-proximal IMUs are shown in the panels below.

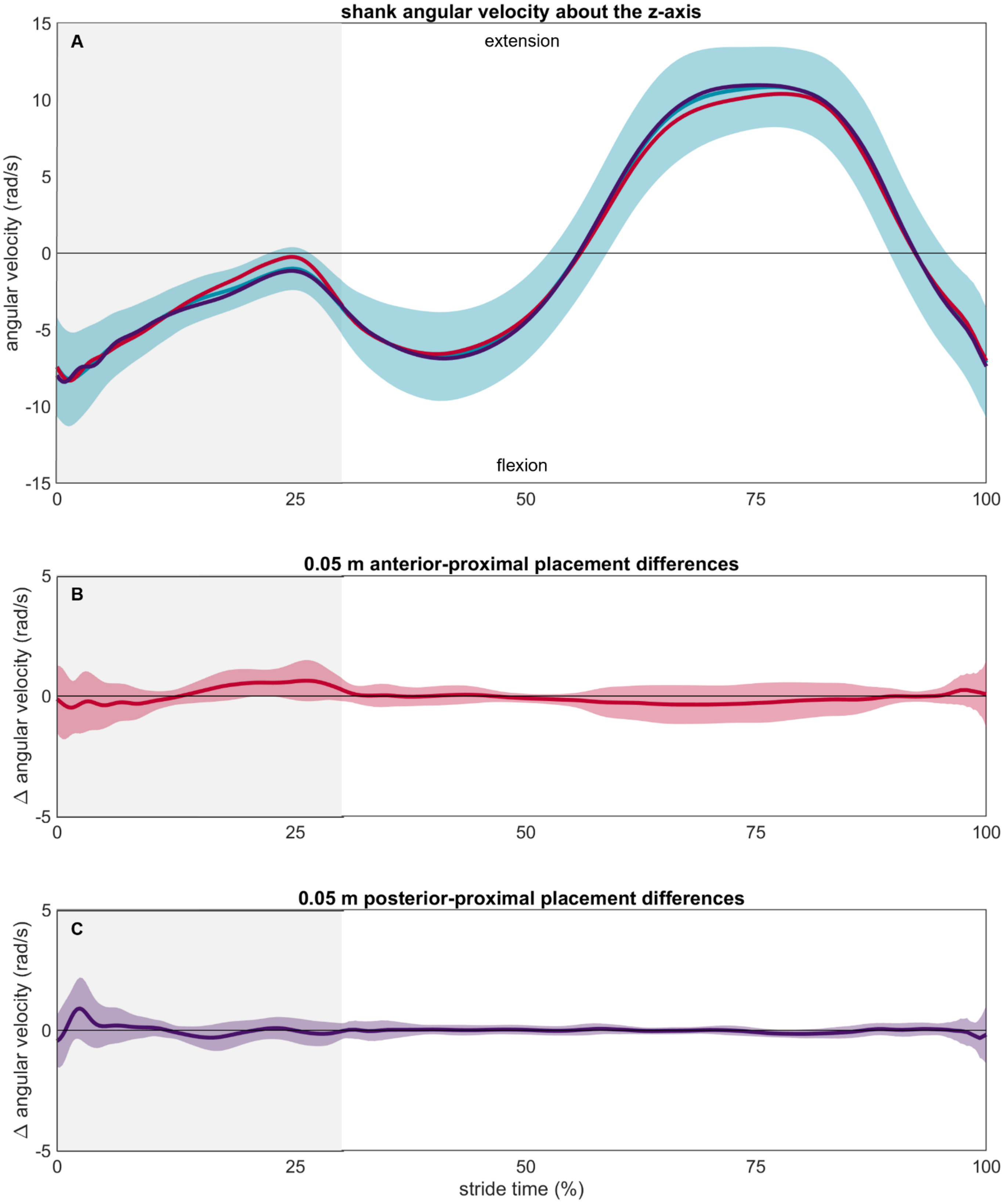

Figure S14: **(A)** Shank z axis angular velocities measured at the reference (blue), anterior-proximal misplaced (red), and posterior-proximal (purple) IMUs. The light blue area represents ± 1 SD about the reference mean. The light grey background represents stance while the white background represents swing. Differences between the reference and **(B)** anterior-proximal and **(C)** posterior-proximal IMUs are shown in the panels below.

B.3. Pelvis acceleration

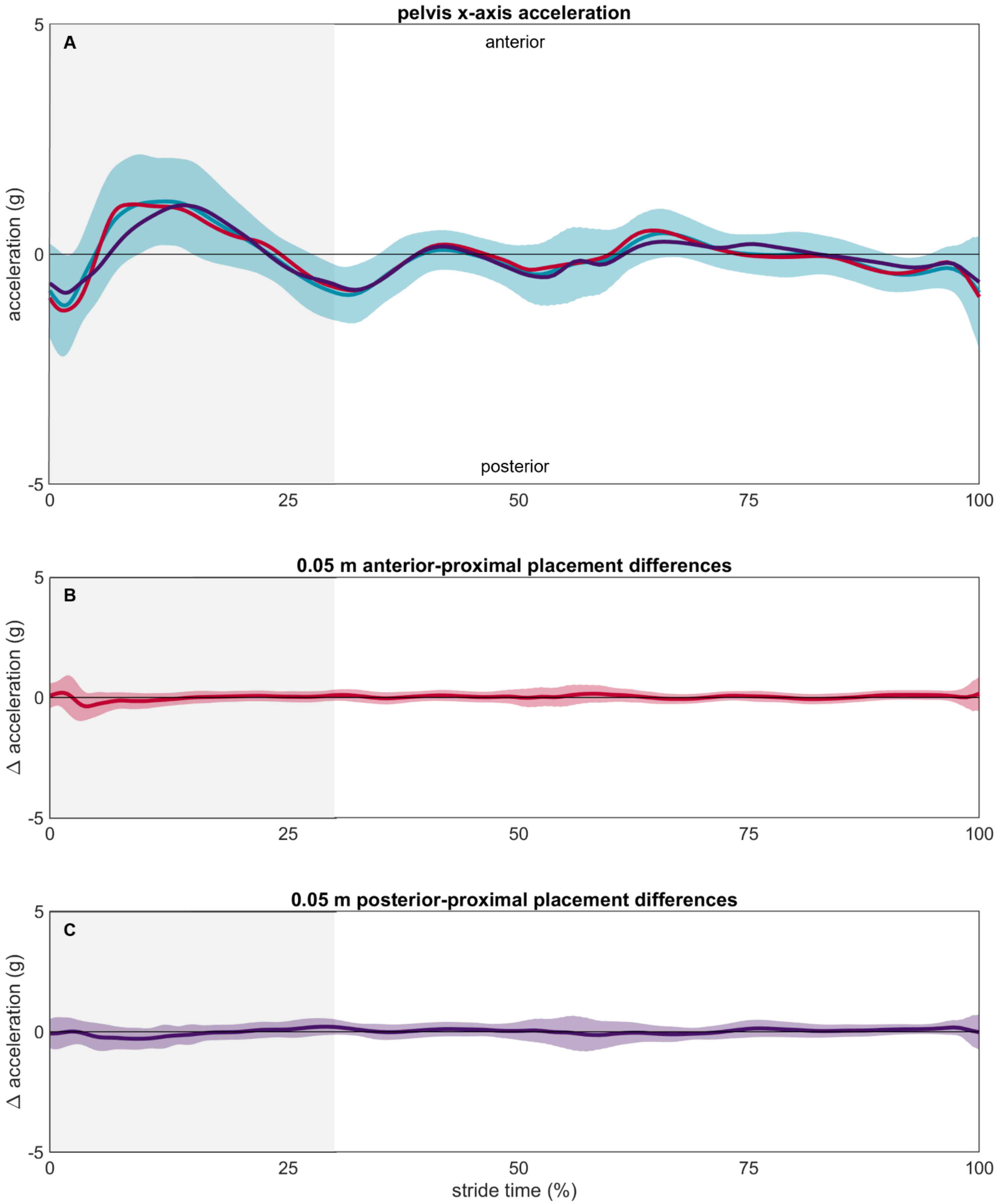

Figure S15: **(A)** Pelvis x axis accelerations measured at the reference (blue), anterior-proximal misplaced (red), and posterior-proximal (purple) IMUs. The light blue area represents ± 1 SD about the reference mean. The light grey background represents stance while the white background represents swing. Differences between the reference and **(B)** anterior-proximal and **(C)** posterior-proximal IMUs are shown in the panels below.

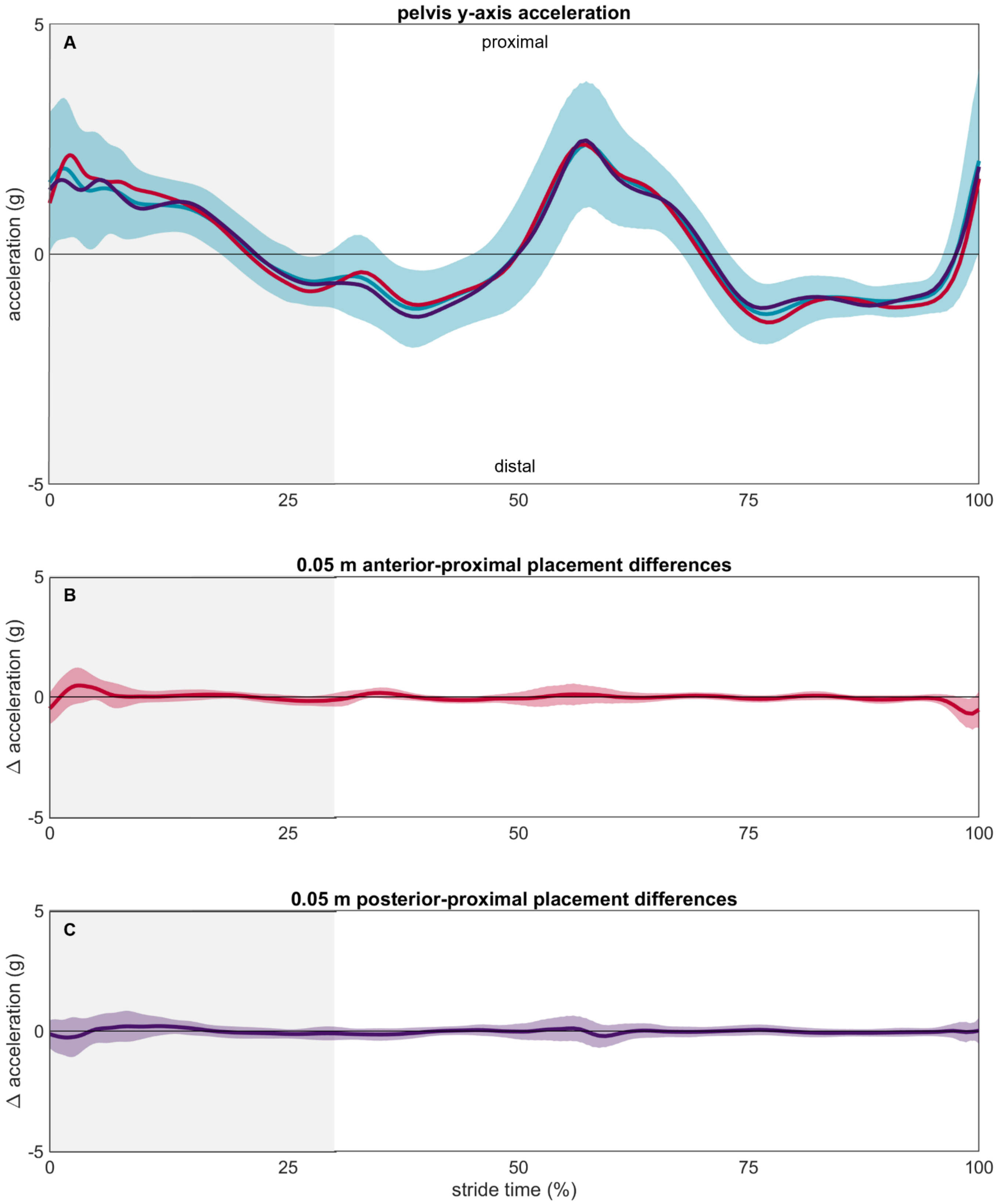

Figure S16: **(A)** Pelvis y axis accelerations measured at the reference (blue), anterior-proximal misplaced (red), and posterior-proximal (purple) IMUs. The light blue area represents ± 1 SD about the reference mean. The light grey background represents stance while the white background represents swing. Differences between the reference and **(B)** anterior-proximal and **(C)** posterior-proximal IMUs are shown in the panels below.

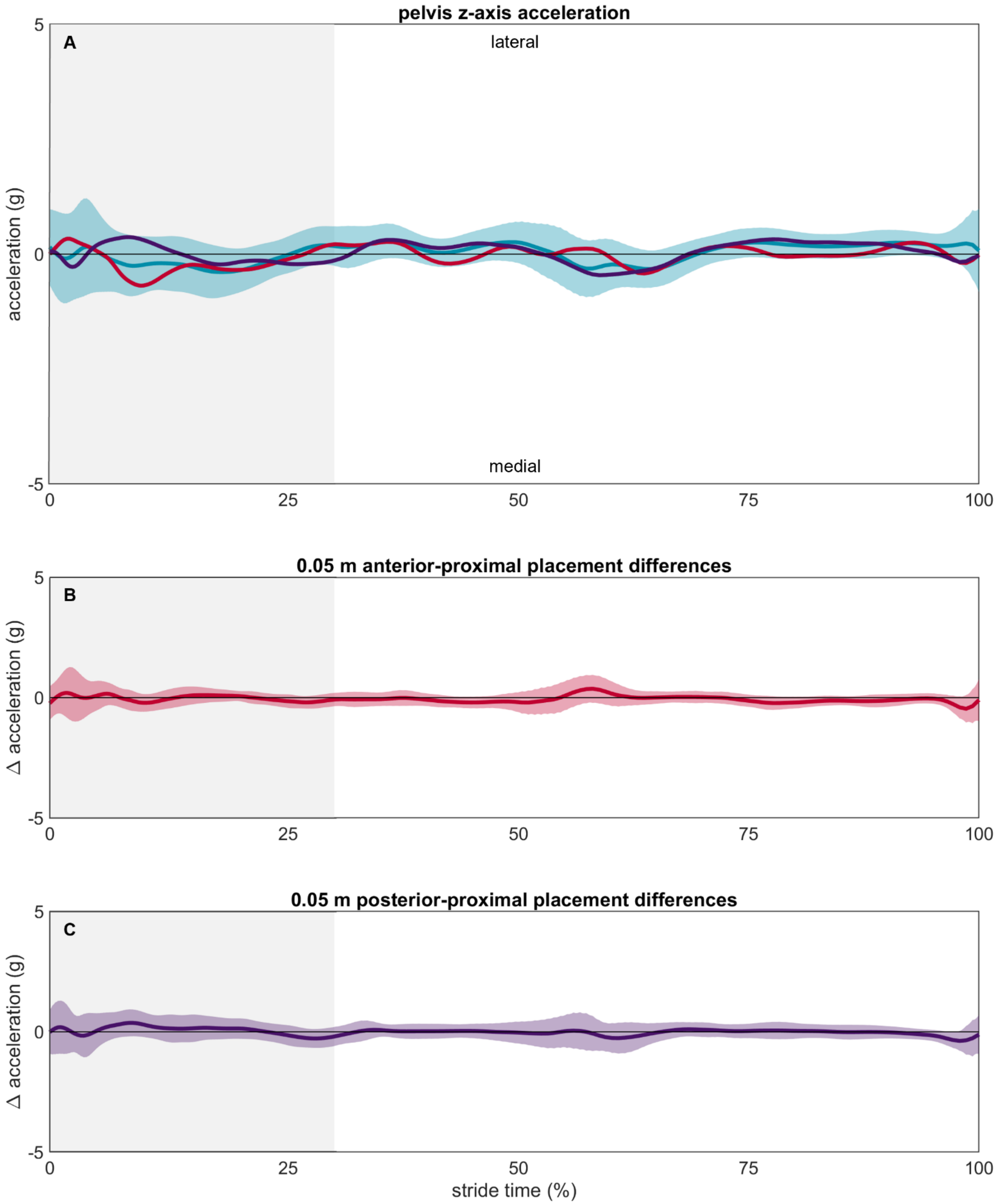

Figure S17: **(A)** Pelvis z axis accelerations measured at the reference (blue), anterior-proximal misplaced (red), and posterior-proximal (purple) IMUs. The light blue area represents ± 1 SD about the reference mean. The light grey background represents stance while the white background represents swing. Differences between the reference and **(B)** anterior-proximal and **(C)** posterior-proximal IMUs are shown in the panels below.

B.4. Pelvis angular velocity

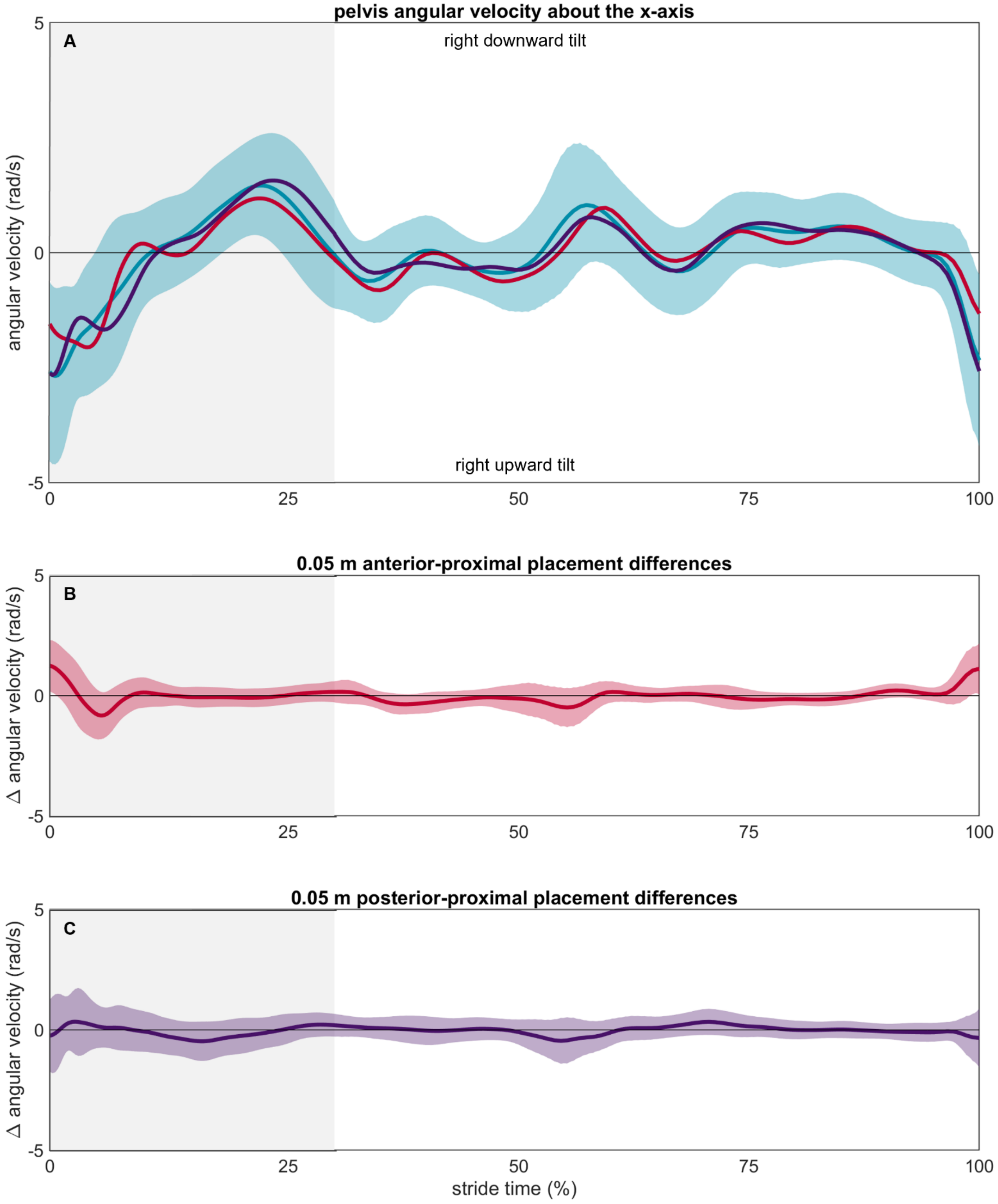

Figure S18: **(A)** Pelvis x axis angular velocities measured at the reference (blue), anterior-proximal misplaced (red), and posterior-proximal (purple) IMUs. The light blue area represents ± 1 SD about the reference mean. The light grey background represents stance while the white background represents swing. Differences between the reference and **(B)** anterior-proximal and **(C)** posterior-proximal IMUs are shown in the panels below.

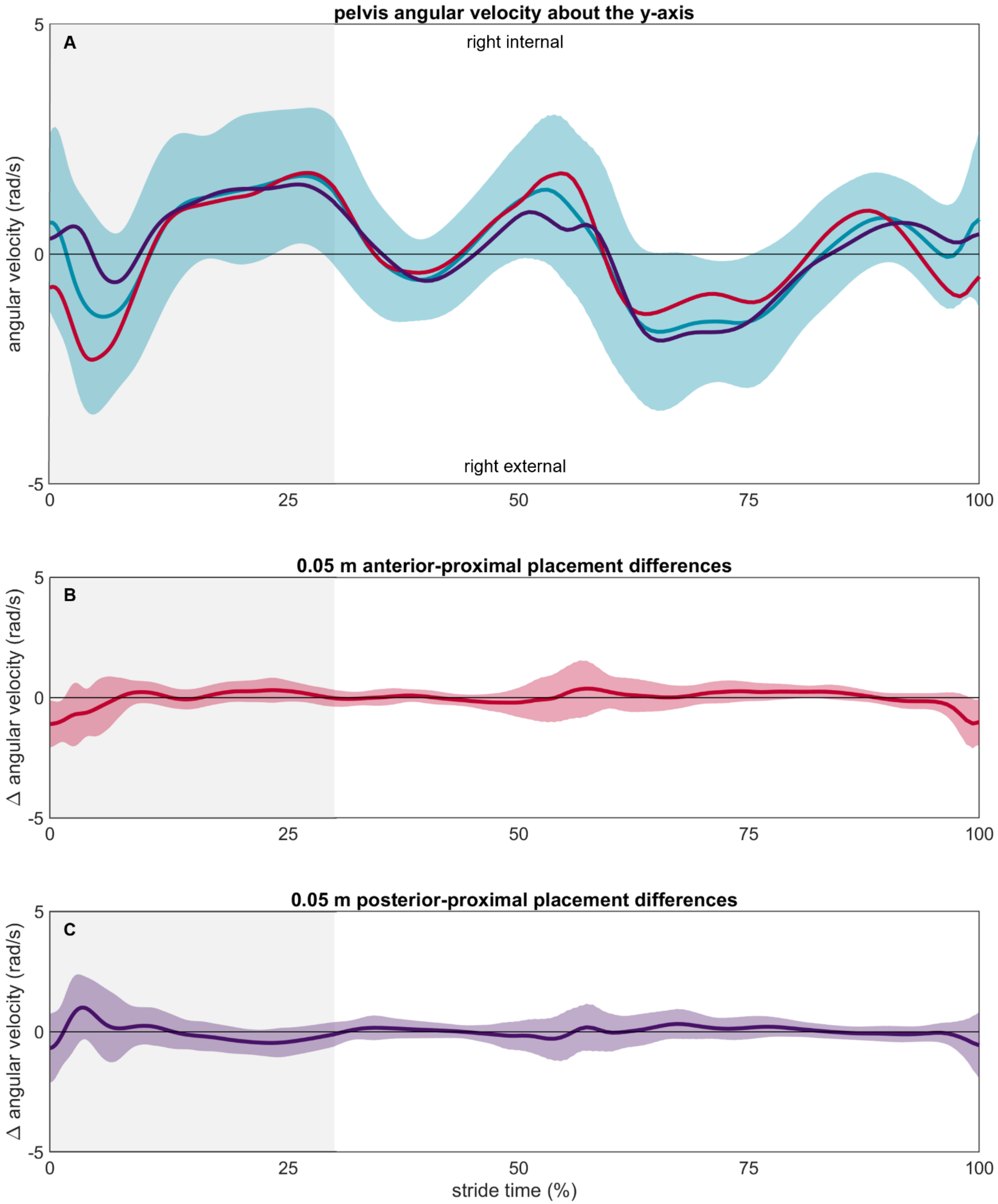

Figure S19: **(A)** Pelvis y axis angular velocities measured at the reference (blue), anterior-proximal misplaced (red), and posterior-proximal (purple) IMUs. The light blue area represents ± 1 SD about the reference mean. The light grey background represents stance while the white background represents swing. Differences between the reference and **(B)** anterior-proximal and **(C)** posterior-proximal IMUs are shown in the panels below.

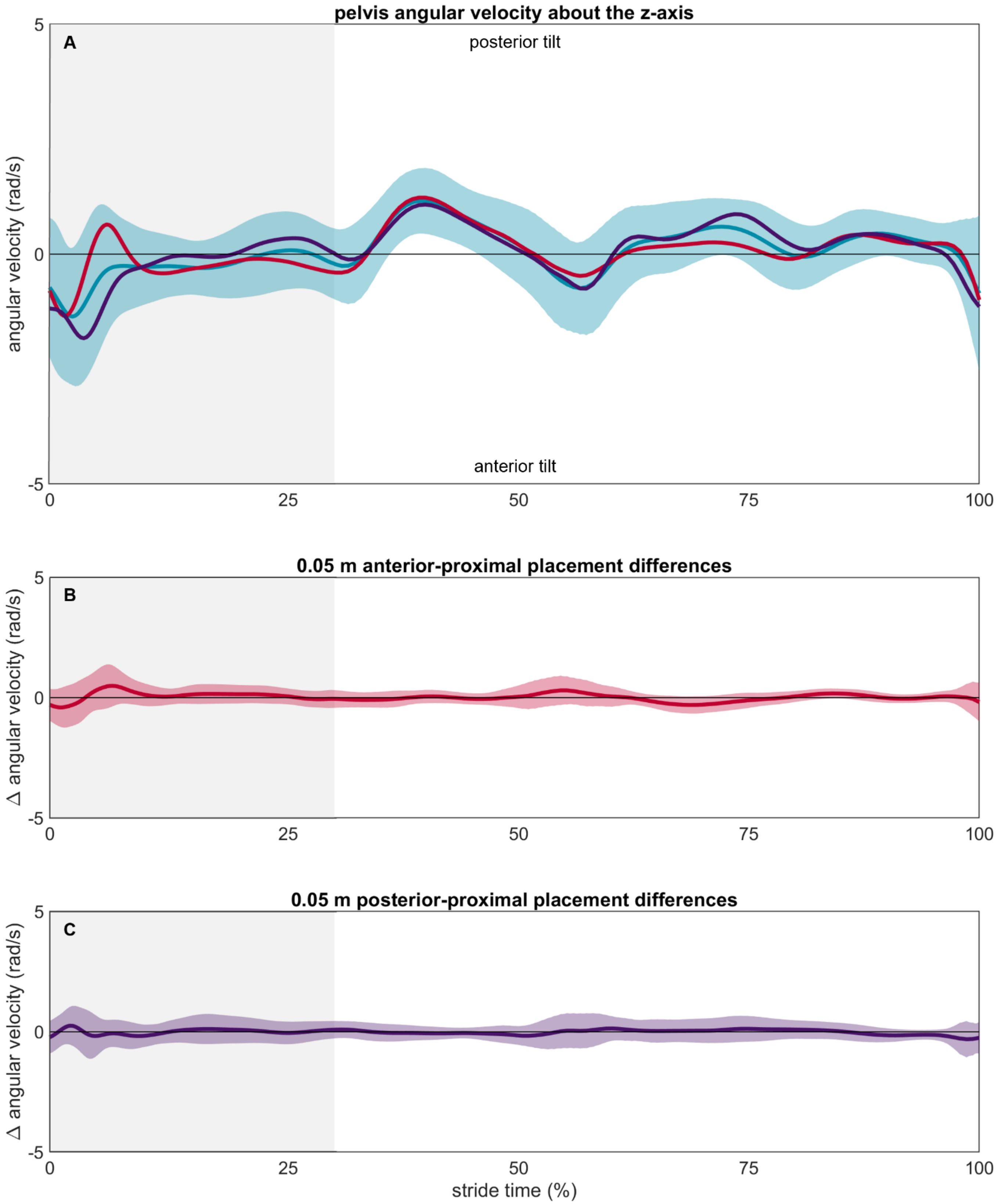

Figure S20: **(A)** Pelvis z axis angular velocities measured at the reference (blue), anterior-proximal misplaced (red), and posterior-proximal (purple) IMUs. The light blue area represents ± 1 SD about the reference mean. The light grey background represents stance while the white background represents swing. Differences between the reference and **(B)** anterior-proximal and **(C)** posterior-proximal IMUs are shown in the panels below.

B.5. Sacrum acceleration

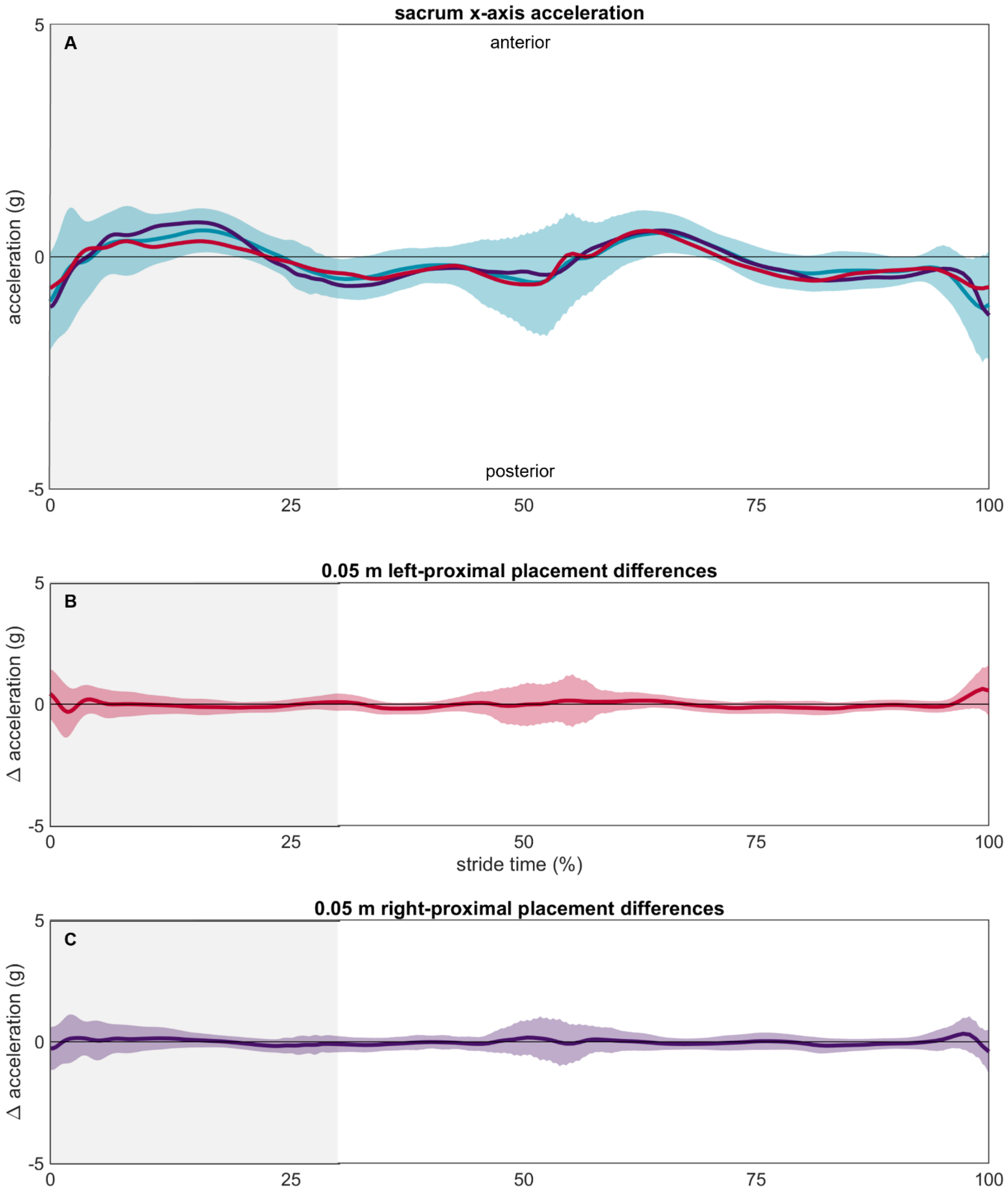

Figure S21: **(A)** Sacrum x axis accelerations measured at the reference (blue), left-proximal misplaced (red), and right-proximal (purple) IMUs. The light blue area represents ± 1 SD about the reference mean. The light grey background represents stance while the white background represents swing. Differences between the reference and **(B)** left-proximal and **(C)** right-proximal IMUs are shown in the panels below.

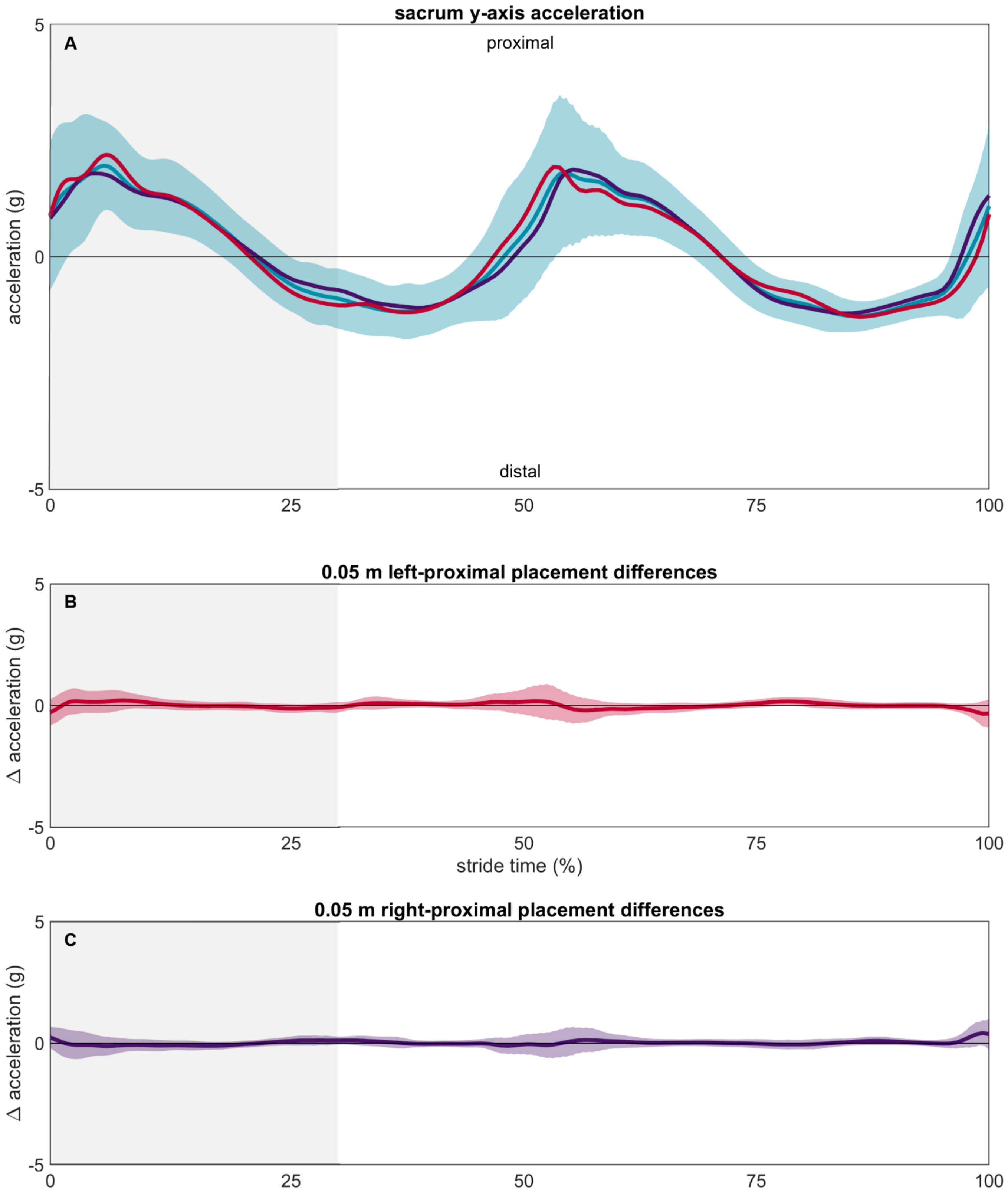

Figure S22: **(A)** Sacrum y axis accelerations measured at the reference (blue), left-proximal misplaced (red), and right-proximal (purple) IMUs. The light blue area represents ± 1 SD about the reference mean. The light grey background represents stance while the white background represents swing. Differences between the reference and **(B)** left-proximal and **(C)** right-proximal IMUs are shown in the panels below.

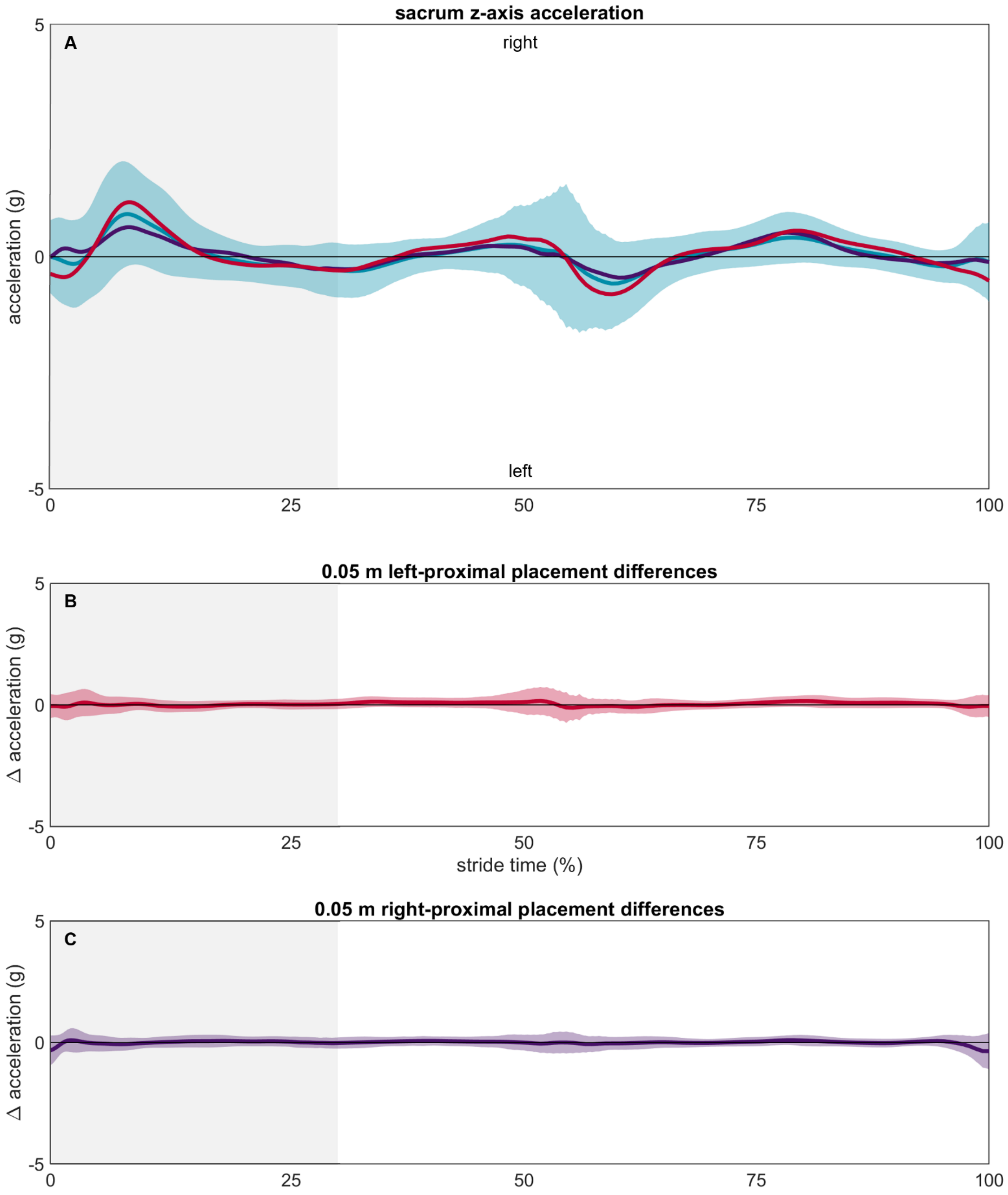

Figure S23: **(A)** Sacrum z axis accelerations measured at the reference (blue), left-proximal misplaced (red), and right-proximal (purple) IMUs. The light blue area represents ± 1 SD about the reference mean. The light grey background represents stance while the white background represents swing. Differences between the reference and **(B)** left-proximal and **(C)** right-proximal IMUs are shown in the panels below.

B.6. Sacrum angular velocity

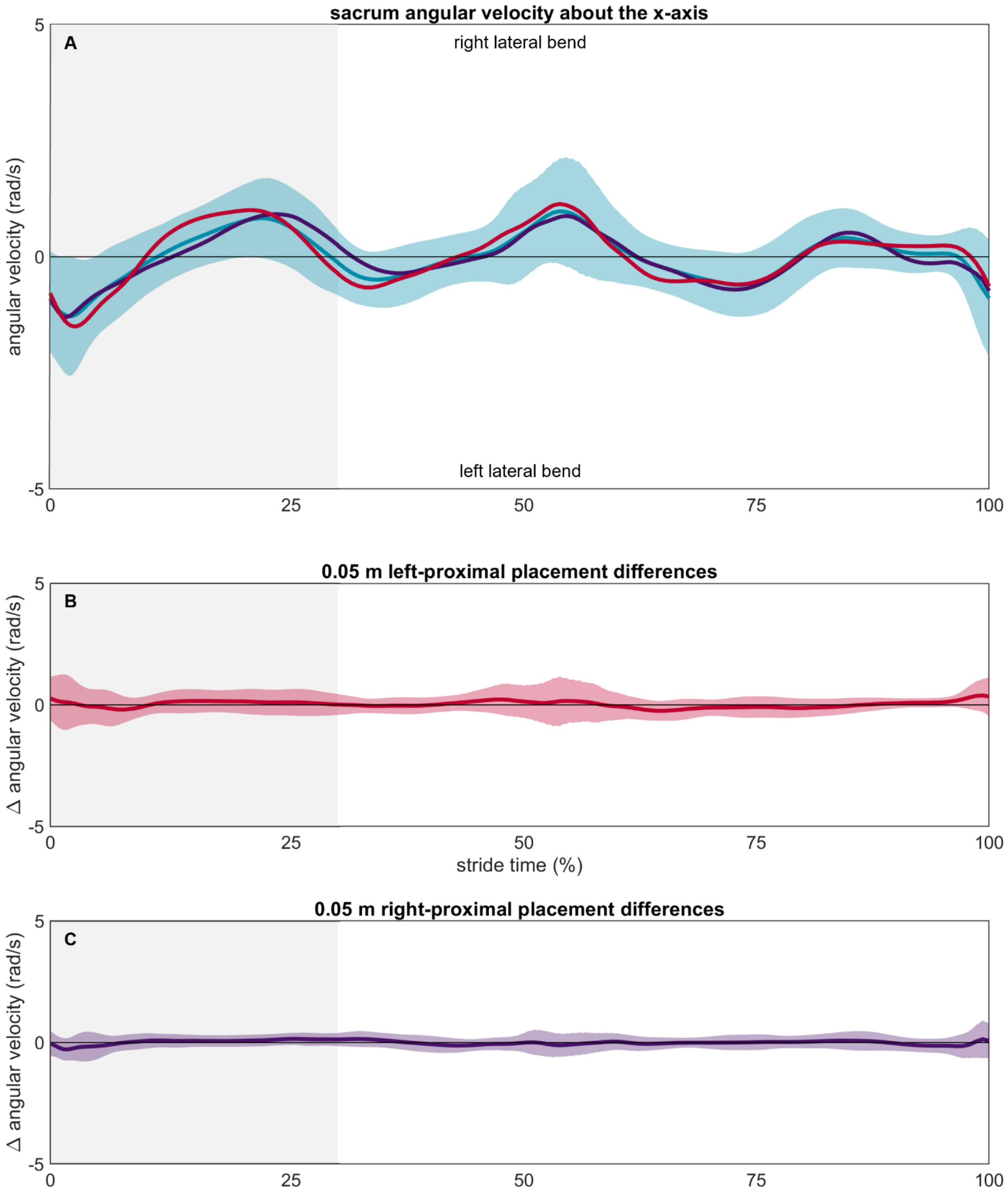

Figure S24: **(A)** Sacrum x axis angular velocity measured at the reference (blue), left-proximal misplaced (red), and right-proximal (purple) IMUs. The light blue area represents ± 1 SD about the reference mean. The light grey background represents stance while the white background represents swing. Differences between the reference and **(B)** left-proximal and **(C)** right-proximal IMUs are shown in the panels below.

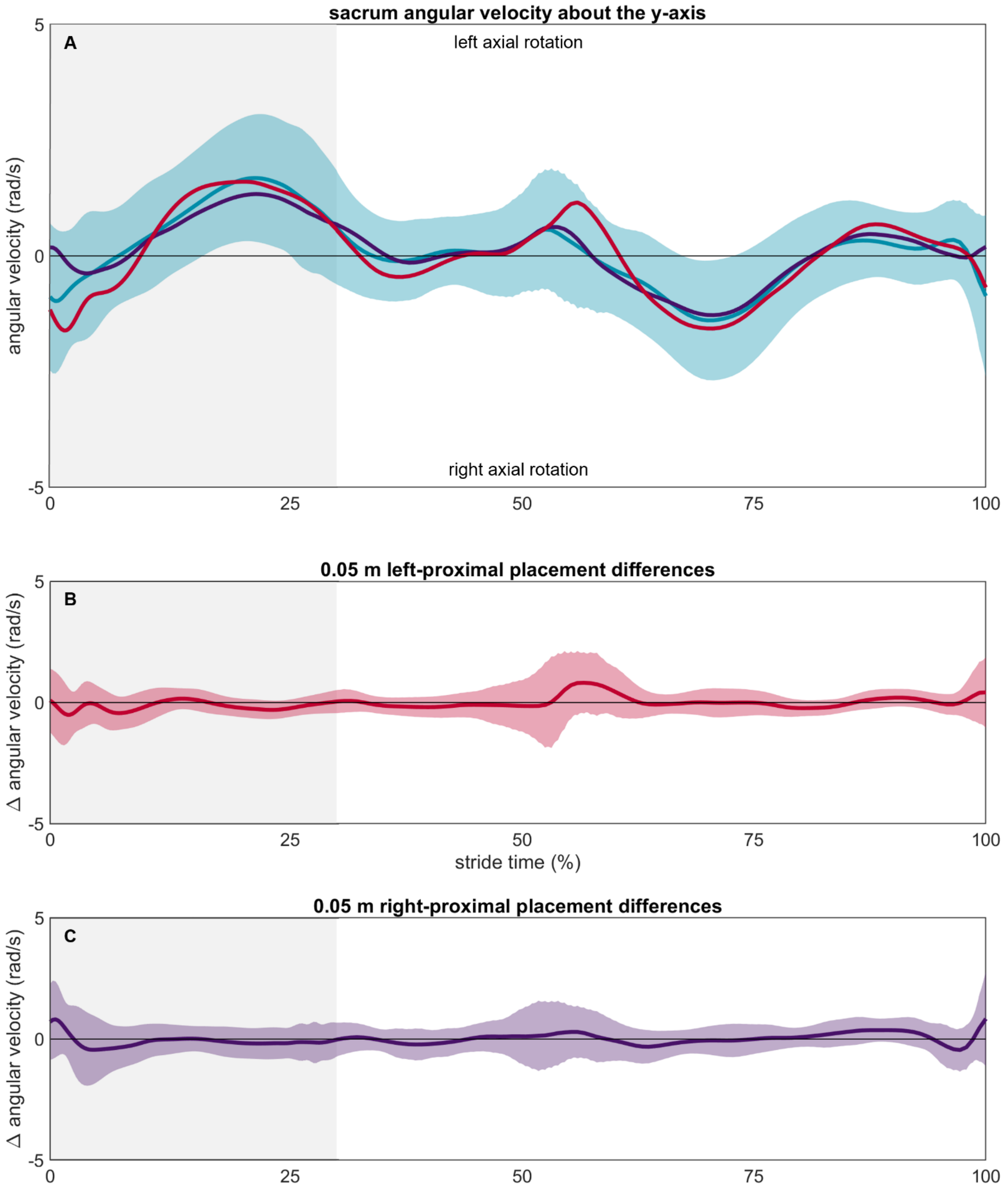

Figure S25: **(A)** Sacrum y axis angular velocity measured at the reference (blue), left-proximal misplaced (red), and right-proximal (purple) IMUs. The light blue area represents ± 1 SD about the reference mean. The light grey background represents stance while the white background represents swing. Differences between the reference and **(B)** left-proximal and **(C)** right-proximal IMUs are shown in the panels below.

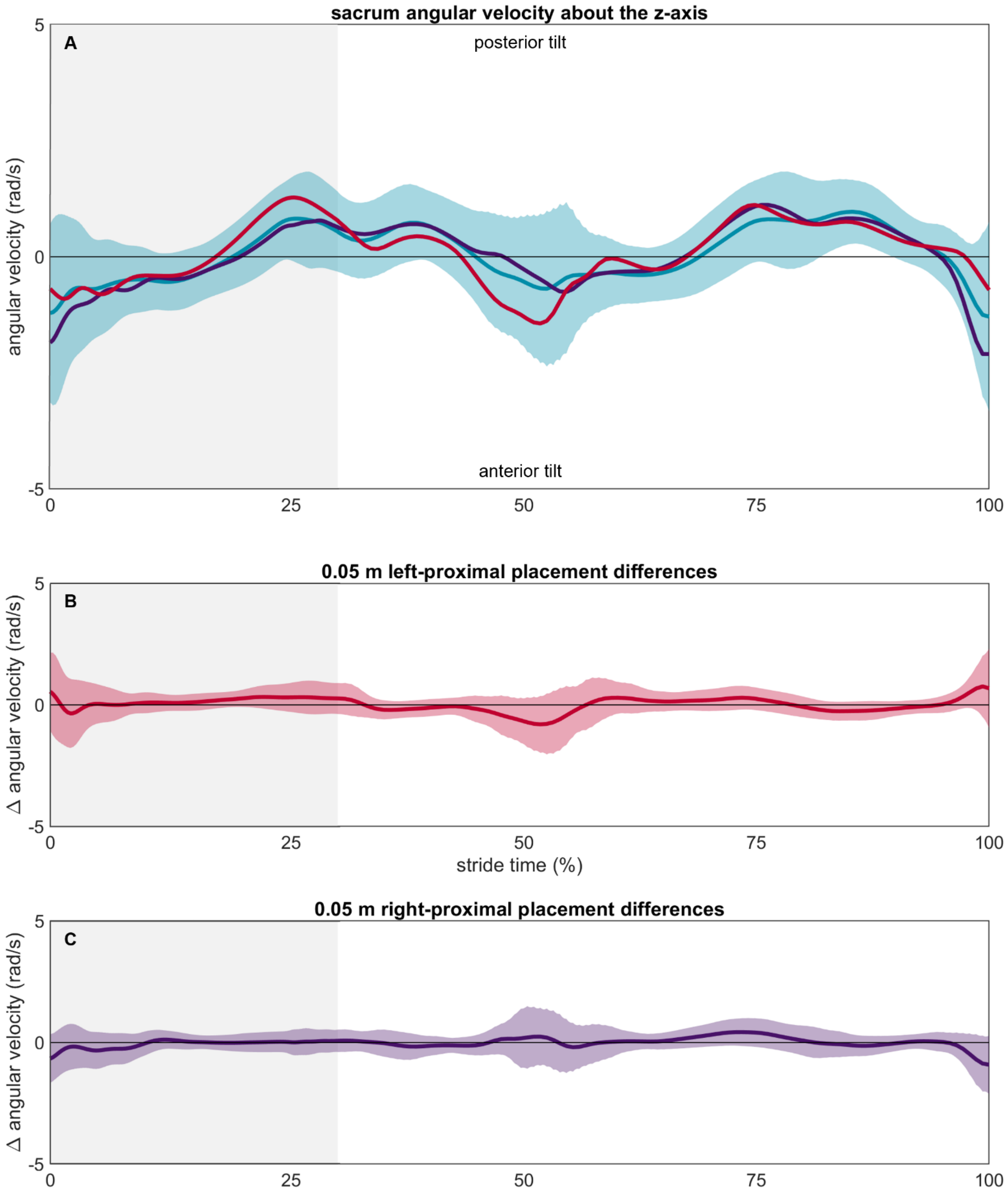

Figure S26: **(A)** Sacrum y axis angular velocity measured at the reference (blue), left-proximal misplaced (red), and right-proximal (purple) IMUs. The light blue area represents ± 1 SD about the reference mean. The light grey background represents stance while the white background represents swing. Differences between the reference and **(B)** left-proximal and **(C)** right-proximal IMUs are shown in the panels below.

C. Frequency domain analyses

Each stride previously segmented in the time domain was zero-padded to the nearest power of two that exceeded the stride duration. The power spectral density from 0 to the Nyquist frequency (500 Hz) was then calculated using a Fast Fourier Transform. Powers and frequencies were interpolated to 1 Hz bins, then power was normalized so that the sum of powers from 0 to 500 Hz was equal to the mean squared amplitude of the time domain. Thus, final units were g^2/Hz or $(rad/s)^2$/Hz [10, 11, 12]. Magnitude-squared coherences between reference and misplaced IMU signals were then calculated via the Welch method. This yields a number between 0 and 1 at each frequency with 0 indicating that the misplaced signal cannot be predicted from the reference signal using a linear model and 1 indicating that the two signals are perfectly related through a linear model. Finally, the proportion of signal power in three equally sized bins from 0 to 50 Hz (0 to 16 Hz, 17 to 33 Hz, and 34 to 50 Hz) was calculated [13].

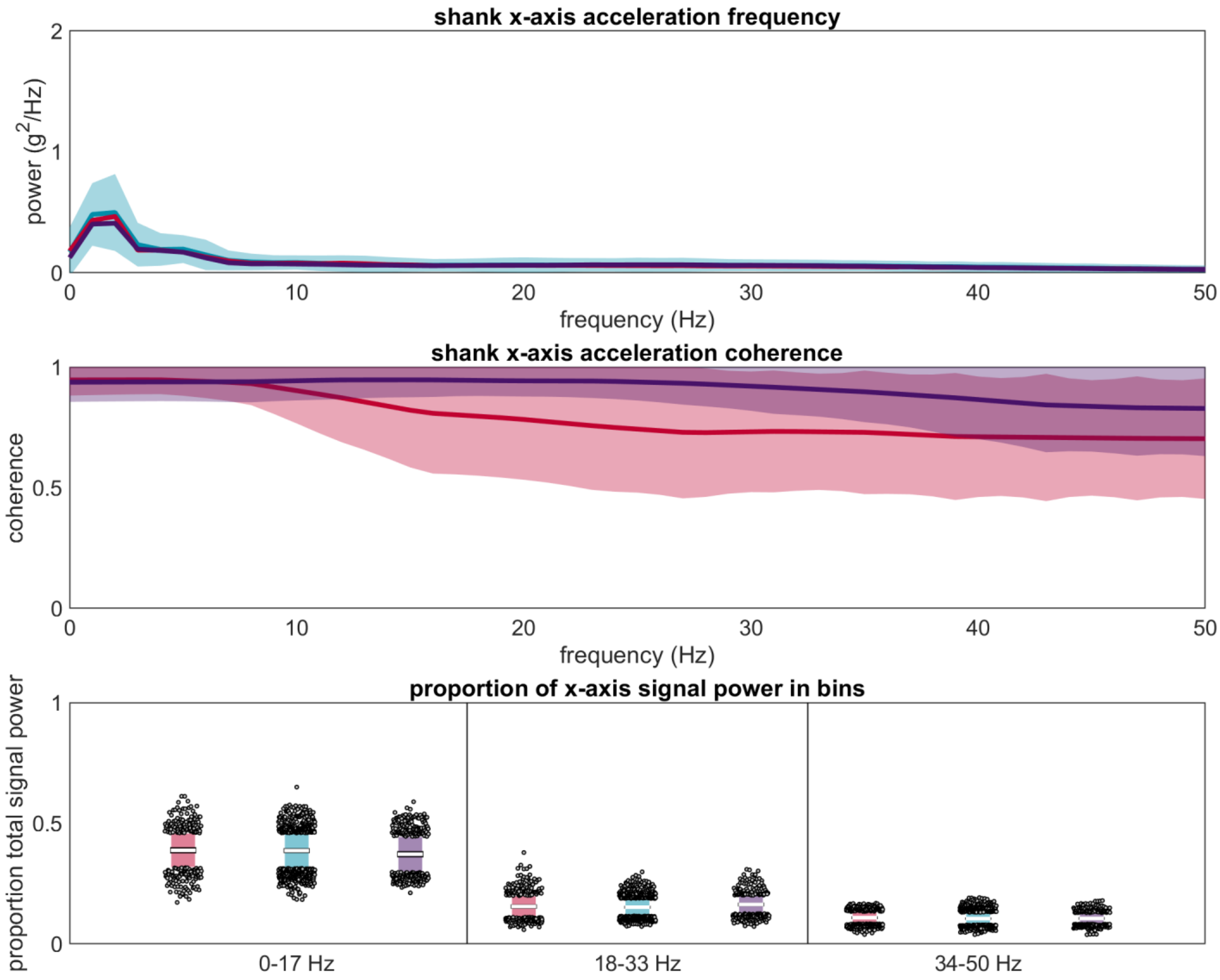

Figure S27: **(Top)** Power spectral density of the shank x axis acceleration from 0 to 50 Hz. The blue line represents the mean for the reference IMU, the blue shaded area represents ± 1 SD, and the red and purple lines represent the anterior-proximal and posterior-proximal misplaced IMUs. **(Middle)** Magnitude squared coherence between each misplaced IMU and the reference IMU. Plotted as mean ± 1 SD. **(Bottom)** Proportion of total signal power observed in the 0-17 Hz, 18-33 Hz, and 34-50 Hz bins. Central white line represents the mean across trials, dark shaded area represents ± 1.96 SEM (confidence interval; difficult to see due to small size), light shaded area represents ± 1 SD, and grey dots represent trials falling outside ± 1 SD.

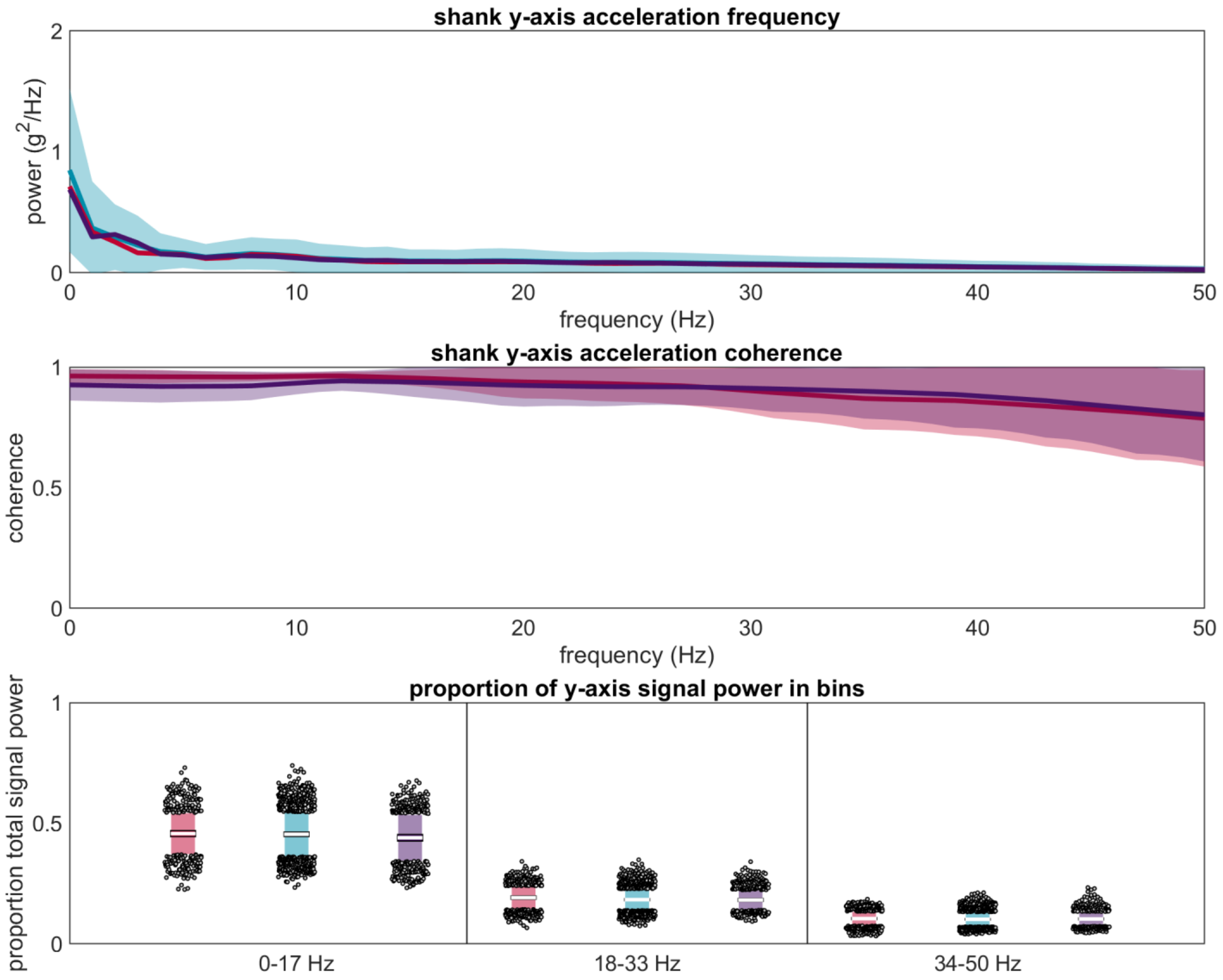

Figure S28: **(Top)** Power spectral density of the shank y axis acceleration from 0 to 50 Hz. The blue line represents the mean for the reference IMU, the blue shaded area represents ± 1 SD, and the red and purple lines represent the anterior-proximal and posterior-proximal misplaced IMUs. **(Middle)** Magnitude squared coherence between each misplaced IMU and the reference IMU. Plotted as mean ± 1 SD. **(Bottom)** Proportion of total signal power observed in the 0-17 Hz, 18-33 Hz, and 34-50 Hz bins. Central white line represents the mean across trials, dark shaded area represents ± 1.96 SEM (confidence interval; difficult to see due to small size), light shaded area represents ± 1 SD, and grey dots represent trials falling outside ± 1 SD.

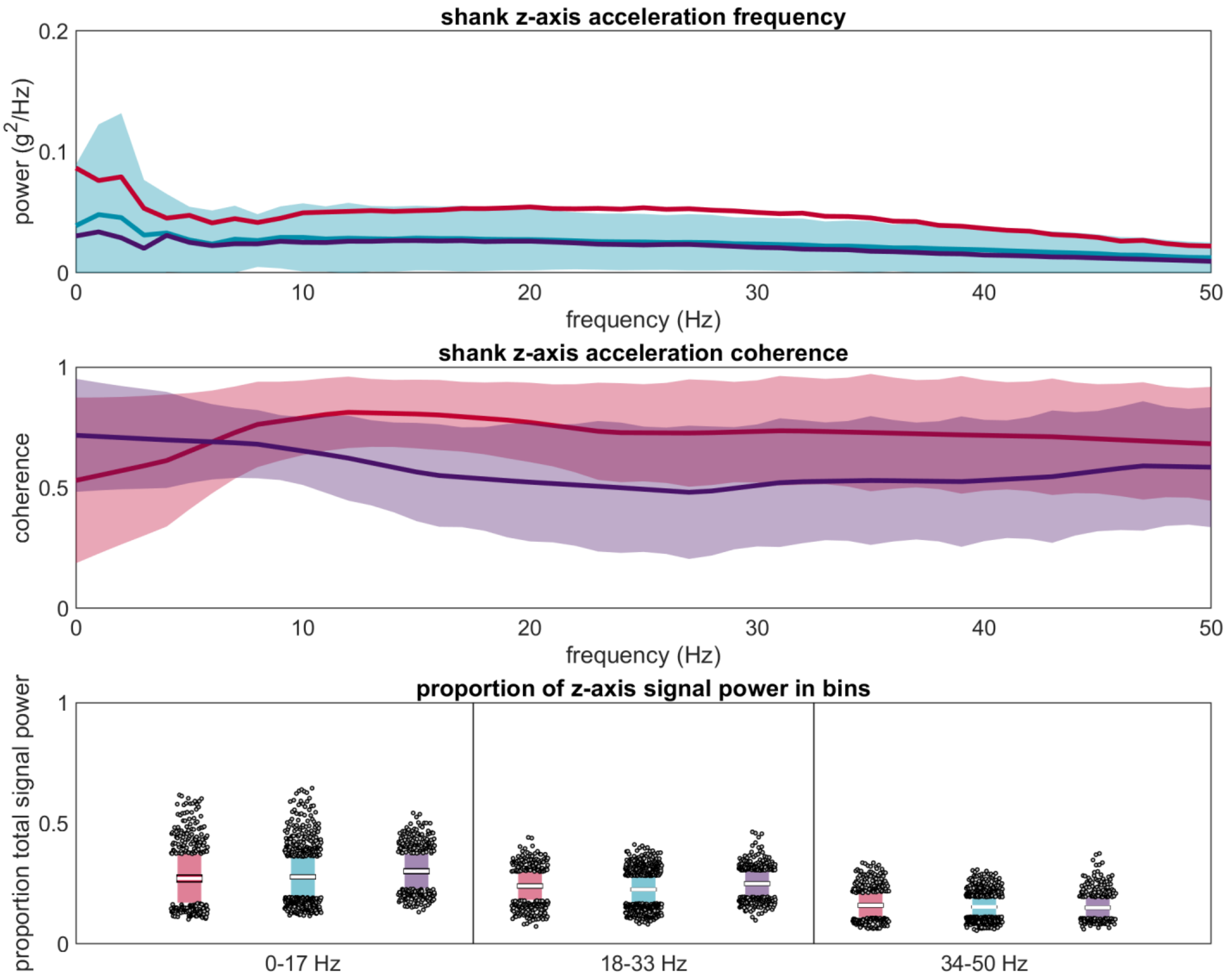

Figure S29: **(Top)** Power spectral density of the shank z axis acceleration from 0 to 50 Hz. The blue line represents the mean for the reference IMU, the blue shaded area represents ± 1 SD, and the red and purple lines represent the anterior-proximal and posterior-proximal misplaced IMUs. **(Middle)** Magnitude squared coherence between each misplaced IMU and the reference IMU. Plotted as mean ± 1 SD. **(Bottom)** Proportion of total signal power observed in the 0-17 Hz, 18-33 Hz, and 34-50 Hz bins. Central white line represents the mean across trials, dark shaded area represents ± 1.96 SEM (confidence interval; difficult to see due to small size), light shaded area represents ± 1 SD, and grey dots represent trials falling outside ± 1 SD.

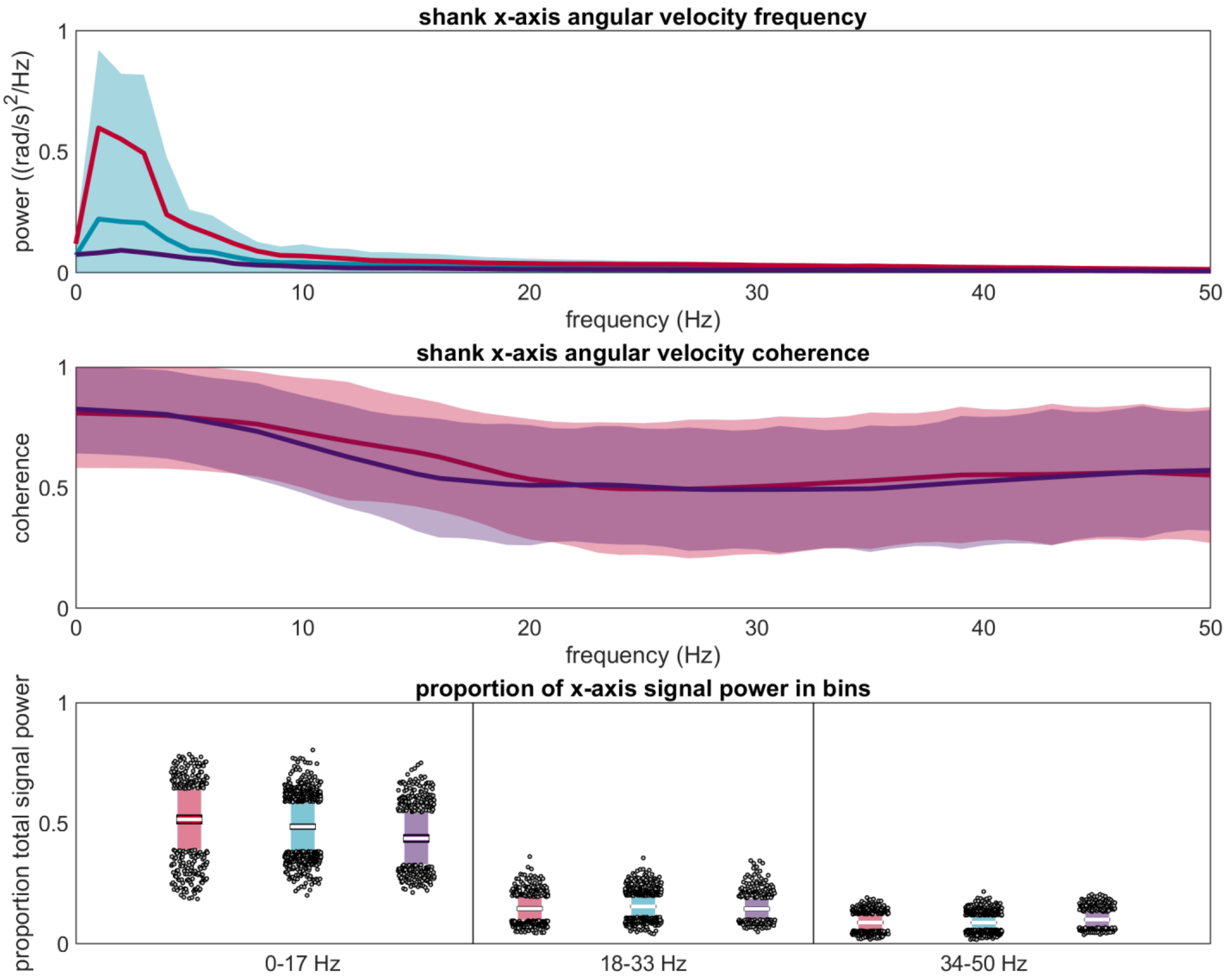

Figure S30: **(Top)** Power spectral density of the shank x axis angular velocity from 0 to 50 Hz. The blue line represents the mean for the reference IMU, the blue shaded area represents ± 1 SD, and the red and purple lines represent the anterior-proximal and posterior-proximal misplaced IMUs. **(Middle)** Magnitude squared coherence between each misplaced IMU and the reference IMU. Plotted as mean ± 1 SD. **(Bottom)** Proportion of total signal power observed in the 0-17 Hz, 18-33 Hz, and 34-50 Hz bins. Central white line represents the mean across trials, dark shaded area represents ± 1.96 SEM (confidence interval; difficult to see due to small size), light shaded area represents ± 1 SD, and grey dots represent trials falling outside ± 1 SD.

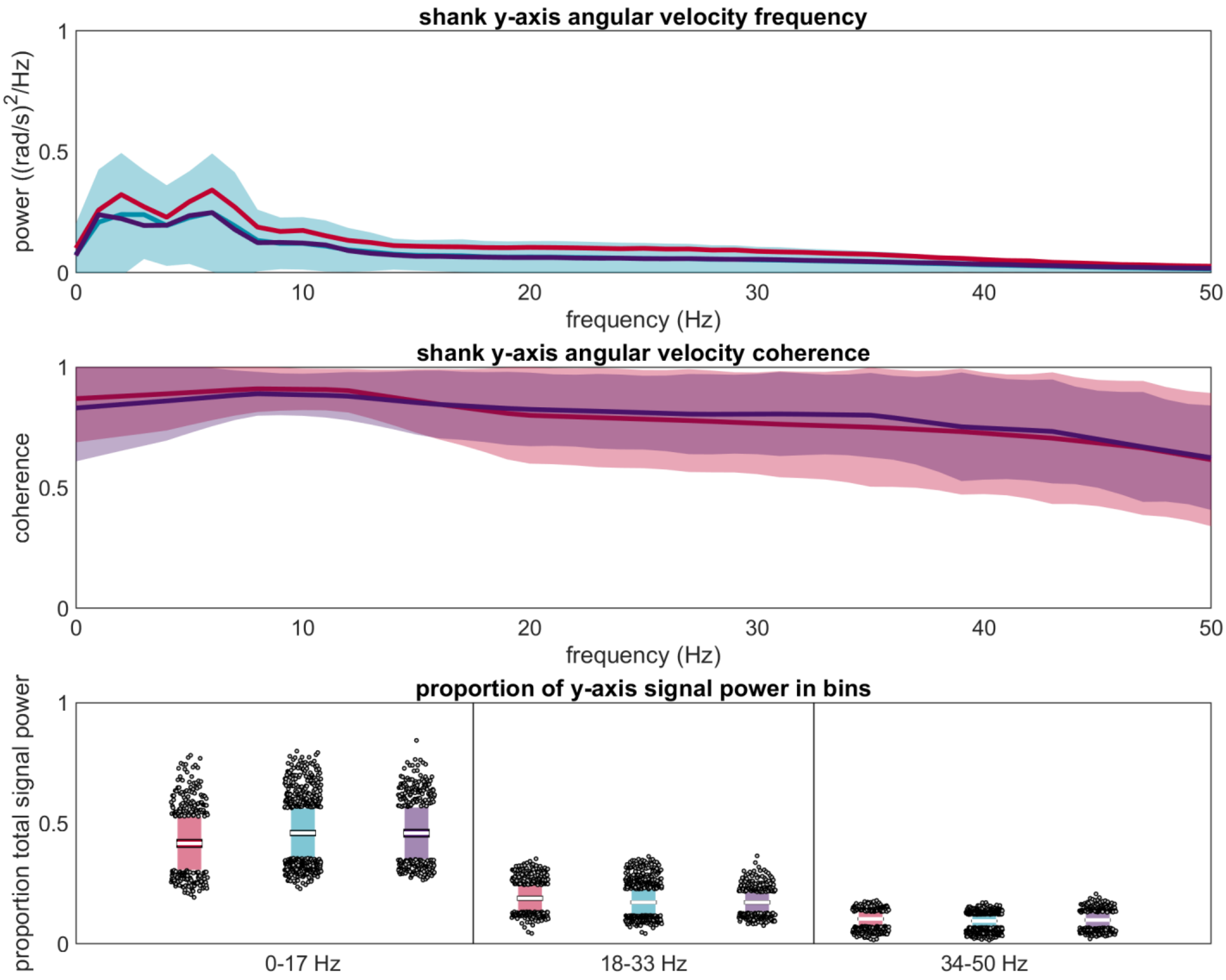

Figure S31: **(Top)** Power spectral density of the shank y axis angular velocity from 0 to 50 Hz. The blue line represents the mean for the reference IMU, the blue shaded area represents ± 1 SD, and the red and purple lines represent the anterior-proximal and posterior-proximal misplaced IMUs. **(Middle)** Magnitude squared coherence between each misplaced IMU and the reference IMU. Plotted as mean ± 1 SD. **(Bottom)** Proportion of total signal power observed in the 0-17 Hz, 18-33 Hz, and 34-50 Hz bins. Central white line represents the mean across trials, dark shaded area represents ± 1.96 SEM (confidence interval; difficult to see due to small size), light shaded area represents ± 1 SD, and grey dots represent trials falling outside ± 1 SD.

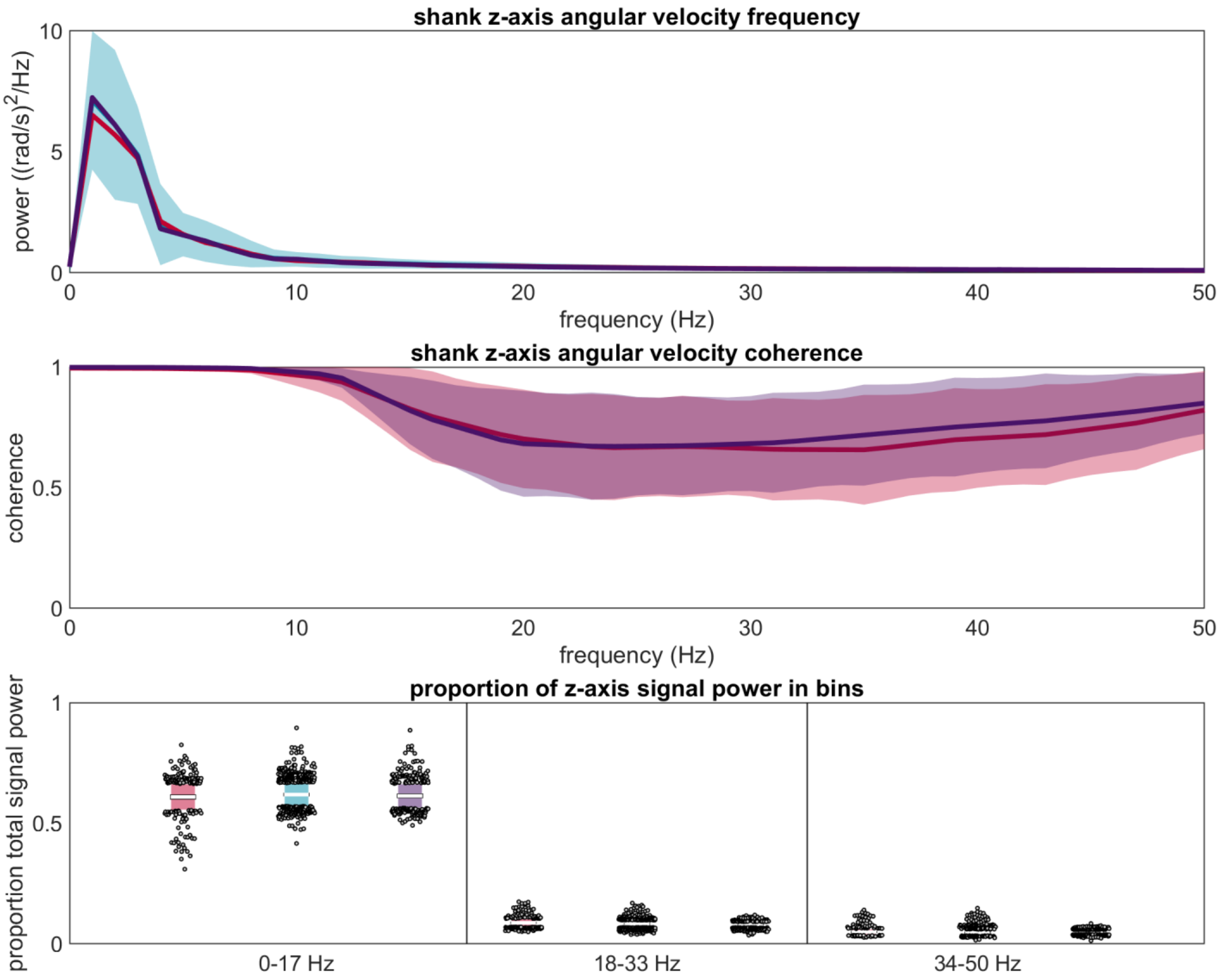

Figure S32: **(Top)** Power spectral density of the shank z axis angular velocity from 0 to 50 Hz. The blue line represents the mean for the reference IMU, the blue shaded area represents ± 1 SD, and the red and purple lines represent the anterior-proximal and posterior-proximal misplaced IMUs. **(Middle)** Magnitude squared coherence between each misplaced IMU and the reference IMU. Plotted as mean ± 1 SD. **(Bottom)** Proportion of total signal power observed in the 0-17 Hz, 18-33 Hz, and 34-50 Hz bins. Central white line represents the mean across trials, dark shaded area represents ± 1.96 SEM (confidence interval; difficult to see due to small size), light shaded area represents ± 1 SD, and grey dots represent trials falling outside ± 1 SD.

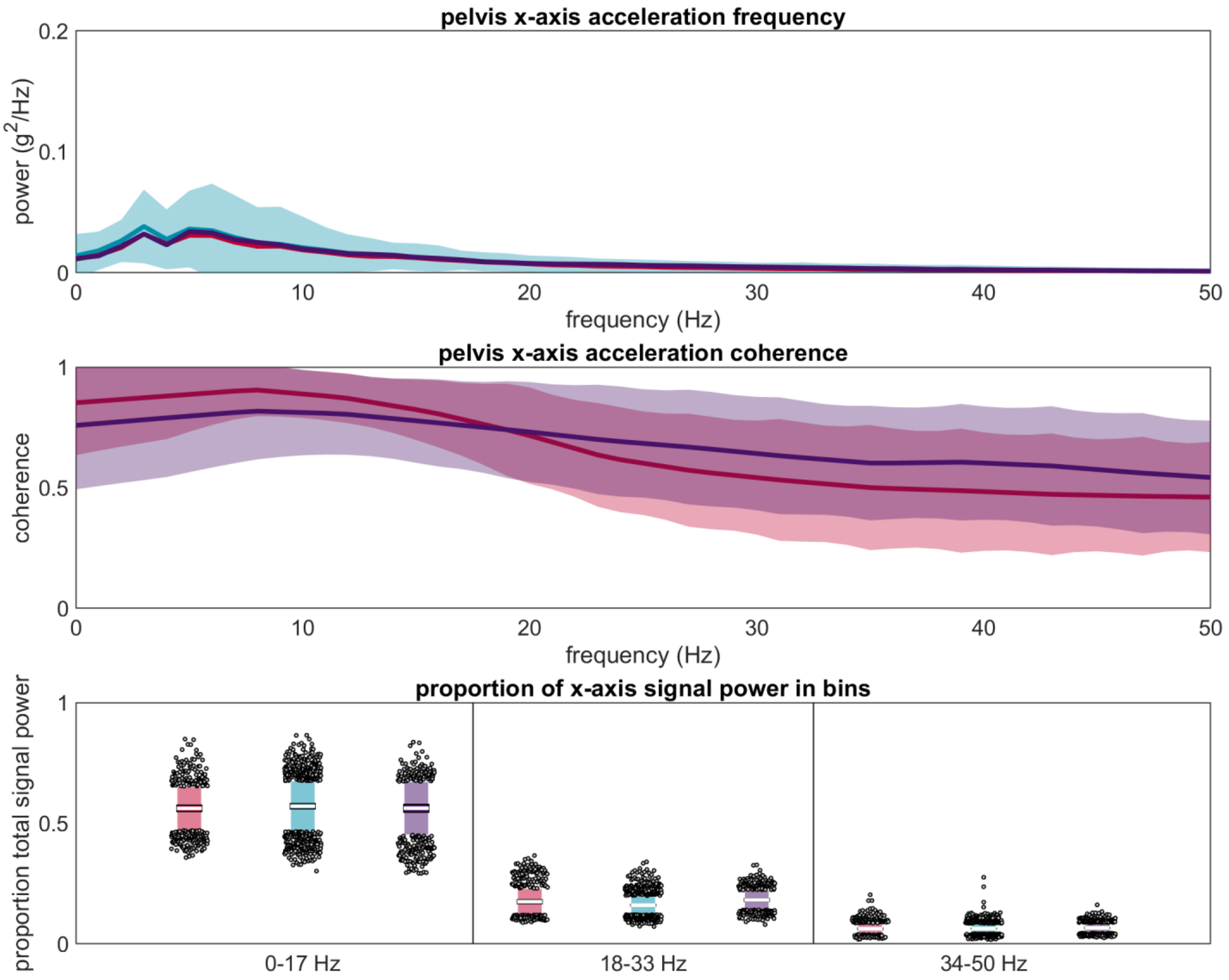

Figure S33: **(Top)** Power spectral density of the pelvis x axis acceleration from 0 to 50 Hz. The blue line represents the mean for the reference IMU, the blue shaded area represents ± 1 SD, and the red and purple lines represent the anterior-proximal and posterior-proximal misplaced IMUs. **(Middle)** Magnitude squared coherence between each misplaced IMU and the reference IMU. Plotted as mean ± 1 SD. **(Bottom)** Proportion of total signal power observed in the 0-17 Hz, 18-33 Hz, and 34-50 Hz bins. Central white line represents the mean across trials, dark shaded area represents ± 1.96 SEM (confidence interval; difficult to see due to small size), light shaded area represents ± 1 SD, and grey dots represent trials falling outside ± 1 SD.

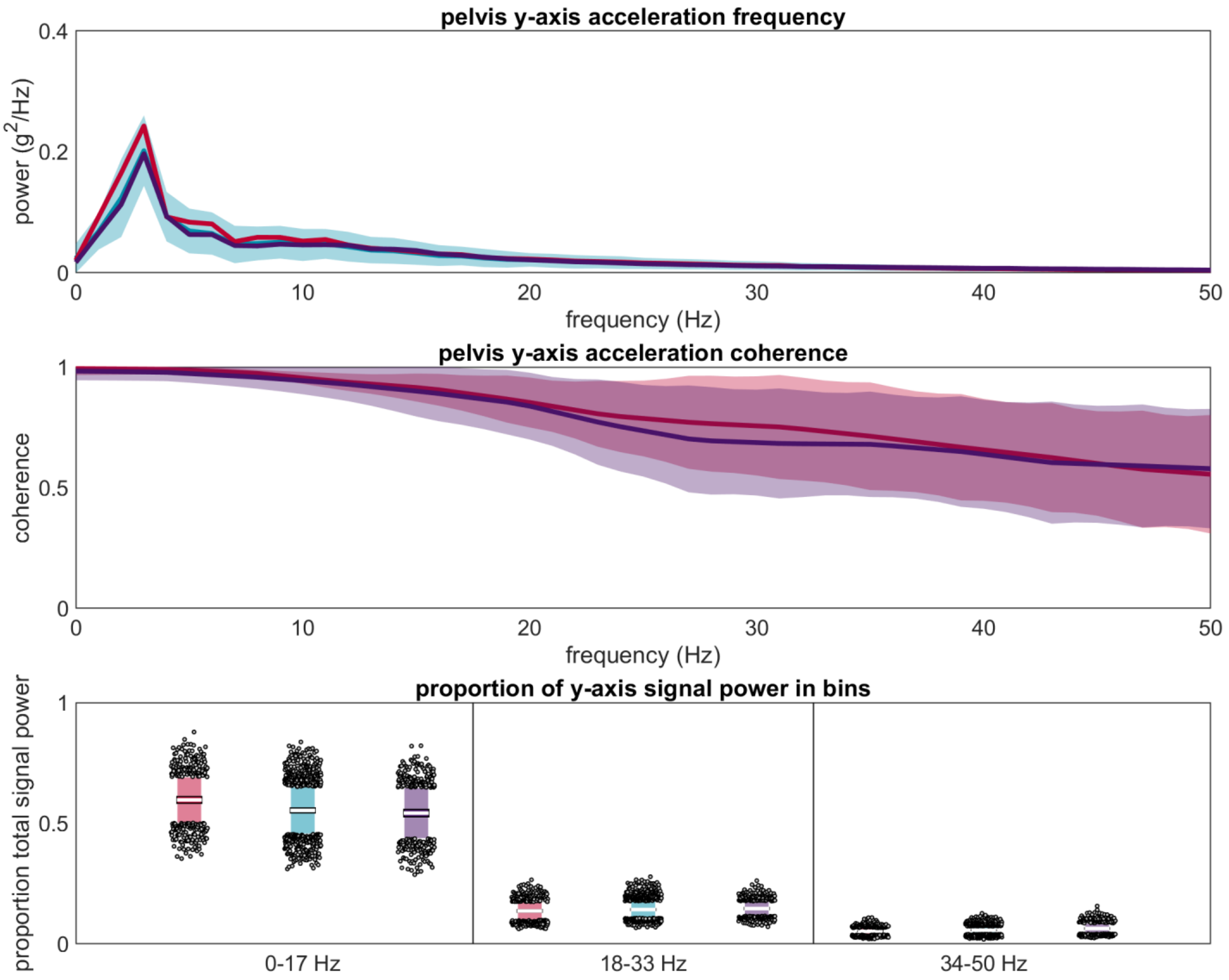

Figure S34: **(Top)** Power spectral density of the pelvis y axis acceleration from 0 to 50 Hz. The blue line represents the mean for the reference IMU, the blue shaded area represents ± 1 SD, and the red and purple lines represent the anterior-proximal and posterior-proximal misplaced IMUs. **(Middle)** Magnitude squared coherence between each misplaced IMU and the reference IMU. Plotted as mean ± 1 SD. **(Bottom)** Proportion of total signal power observed in the 0-17 Hz, 18-33 Hz, and 34-50 Hz bins. Central white line represents the mean across trials, dark shaded area represents ± 1.96 SEM (confidence interval; difficult to see due to small size), light shaded area represents ± 1 SD, and grey dots represent trials falling outside ± 1 SD.

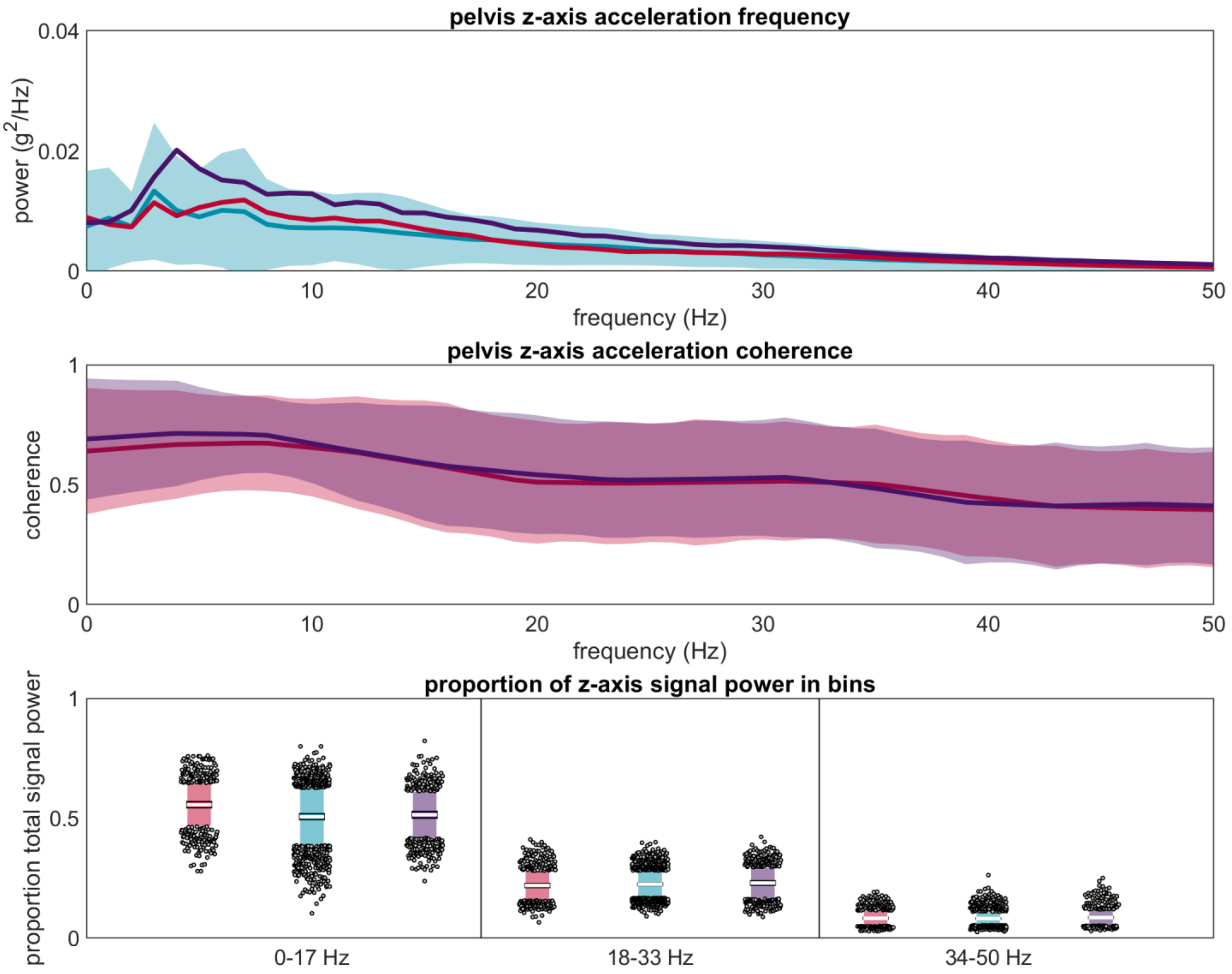

Figure S35: **(Top)** Power spectral density of the pelvis z axis acceleration from 0 to 50 Hz. The blue line represents the mean for the reference IMU, the blue shaded area represents ± 1 SD, and the red and purple lines represent the anterior-proximal and posterior-proximal misplaced IMUs. **(Middle)** Magnitude squared coherence between each misplaced IMU and the reference IMU. Plotted as mean ± 1 SD. **(Bottom)** Proportion of total signal power observed in the 0-17 Hz, 18-33 Hz, and 34-50 Hz bins. Central white line represents the mean across trials, dark shaded area represents ± 1.96 SEM (confidence interval; difficult to see due to small size), light shaded area represents ± 1 SD, and grey dots represent trials falling outside ± 1 SD.

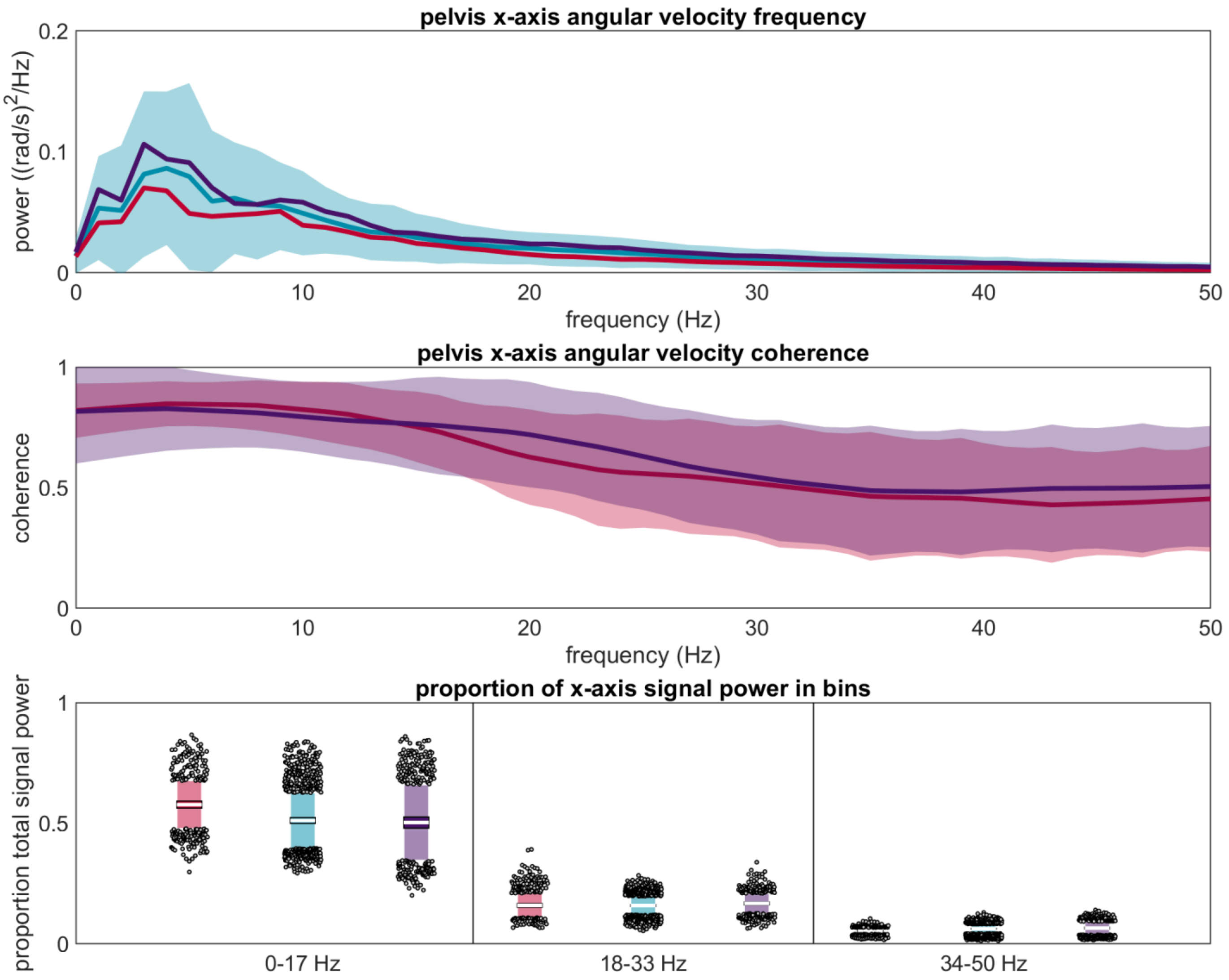

Figure S36: **(Top)** Power spectral density of the pelvis x axis angular velocity from 0 to 50 Hz. The blue line represents the mean for the reference IMU, the blue shaded area represents ± 1 SD, and the red and purple lines represent the anterior-proximal and posterior-proximal misplaced IMUs. **(Middle)** Magnitude squared coherence between each misplaced IMU and the reference IMU. Plotted as mean ± 1 SD. **(Bottom)** Proportion of total signal power observed in the 0-17 Hz, 18-33 Hz, and 34-50 Hz bins. Central white line represents the mean across trials, dark shaded area represents ± 1.96 SEM (confidence interval; difficult to see due to small size), light shaded area represents ± 1 SD, and grey dots represent trials falling outside ± 1 SD.

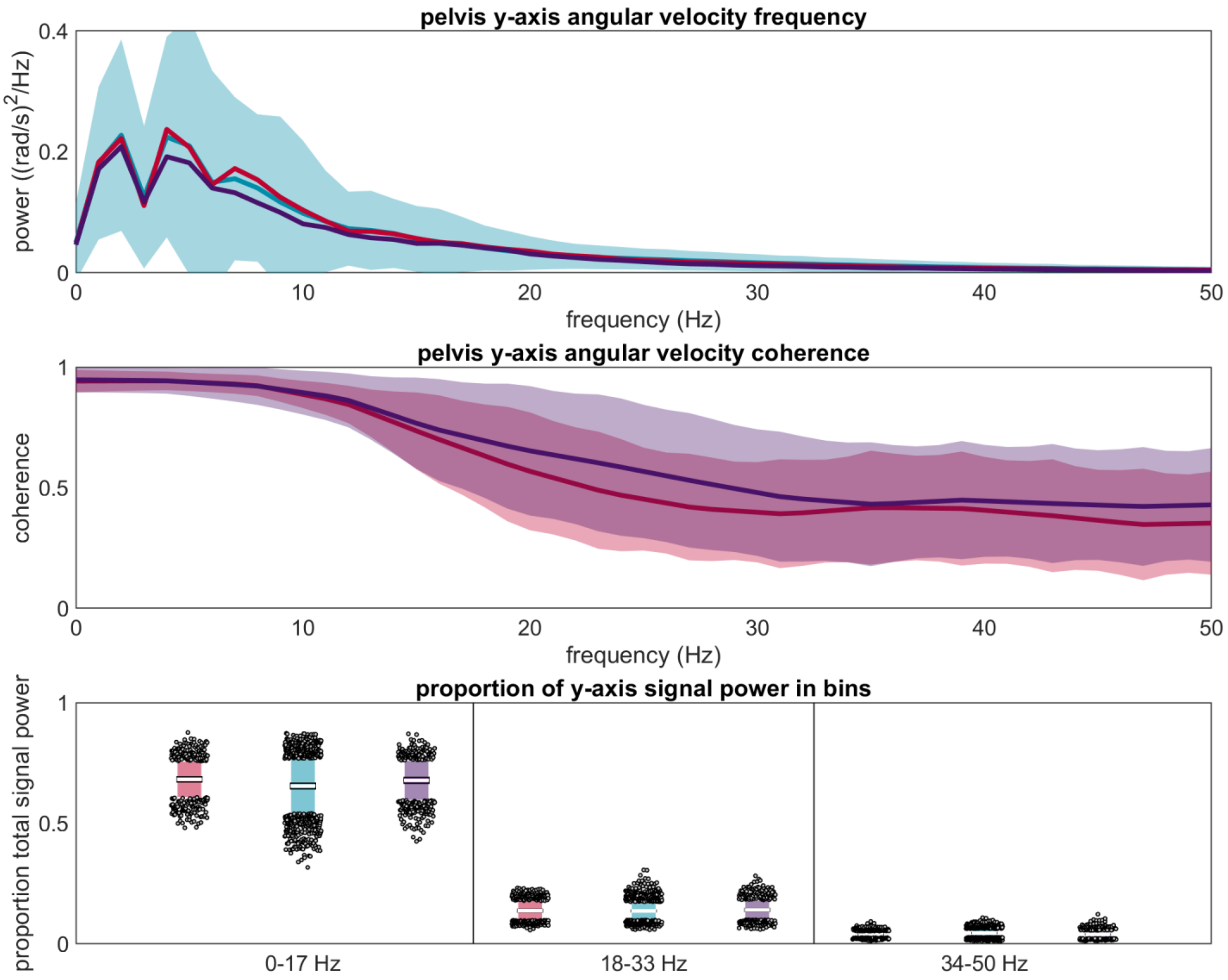

Figure S37: **(Top)** Power spectral density of the pelvis y axis angular velocity from 0 to 50 Hz. The blue line represents the mean for the reference IMU, the blue shaded area represents ± 1 SD, and the red and purple lines represent the anterior-proximal and posterior-proximal misplaced IMUs. **(Middle)** Magnitude squared coherence between each misplaced IMU and the reference IMU. Plotted as mean ± 1 SD. **(Bottom)** Proportion of total signal power observed in the 0-17 Hz, 18-33 Hz, and 34-50 Hz bins. Central white line represents the mean across trials, dark shaded area represents ± 1.96 SEM (confidence interval; difficult to see due to small size), light shaded area represents ± 1 SD, and grey dots represent trials falling outside ± 1 SD.

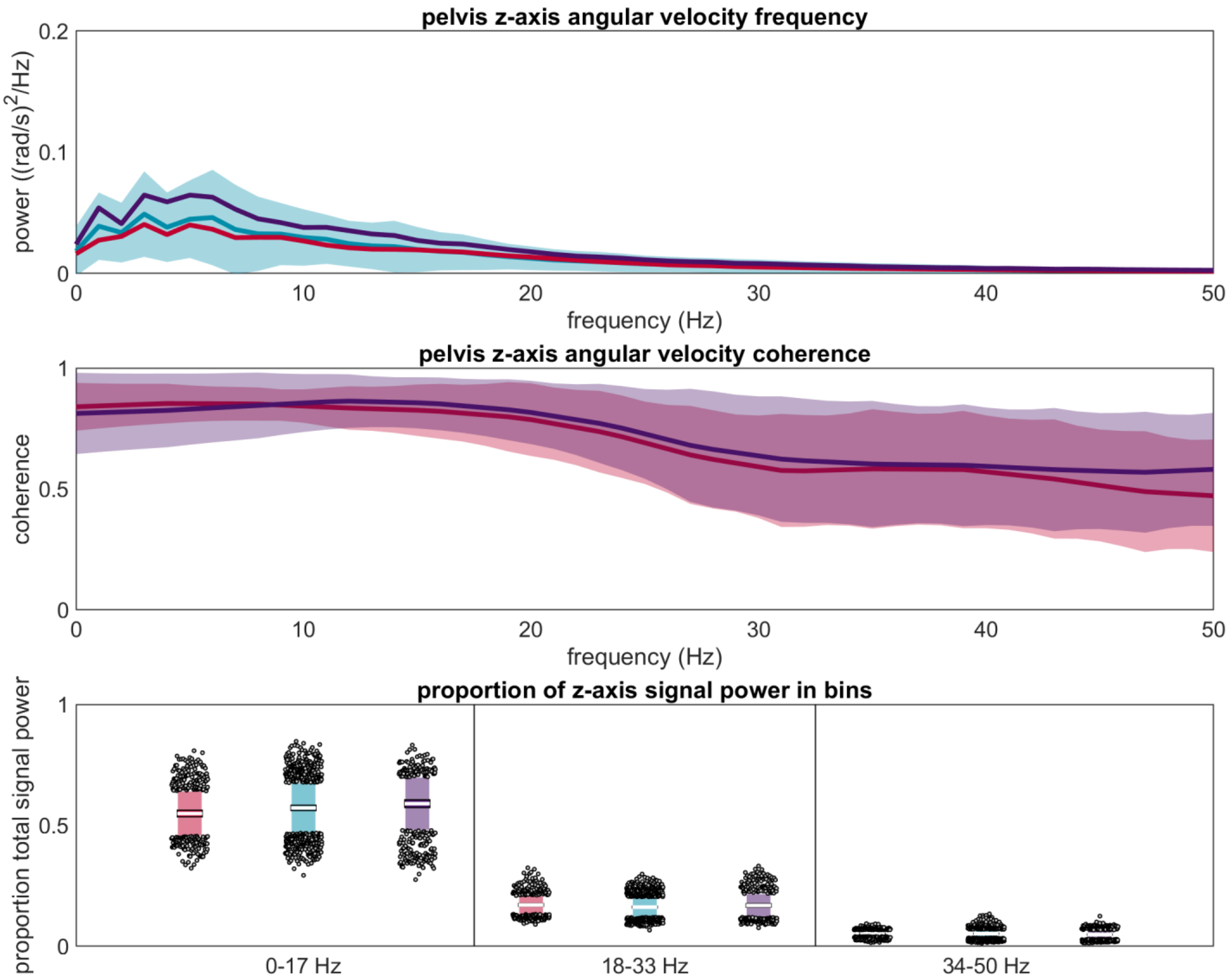

Figure S38: **(Top)** Power spectral density of the pelvis z axis angular velocity from 0 to 50 Hz. The blue line represents the mean for the reference IMU, the blue shaded area represents ± 1 SD, and the red and purple lines represent the anterior-proximal and posterior-proximal misplaced IMUs. **(Middle)** Magnitude squared coherence between each misplaced IMU and the reference IMU. Plotted as mean ± 1 SD. **(Bottom)** Proportion of total signal power observed in the 0-17 Hz, 18-33 Hz, and 34-50 Hz bins. Central white line represents the mean across trials, dark shaded area represents ± 1.96 SEM (confidence interval; difficult to see due to small size), light shaded area represents ± 1 SD, and grey dots represent trials falling outside ± 1 SD.

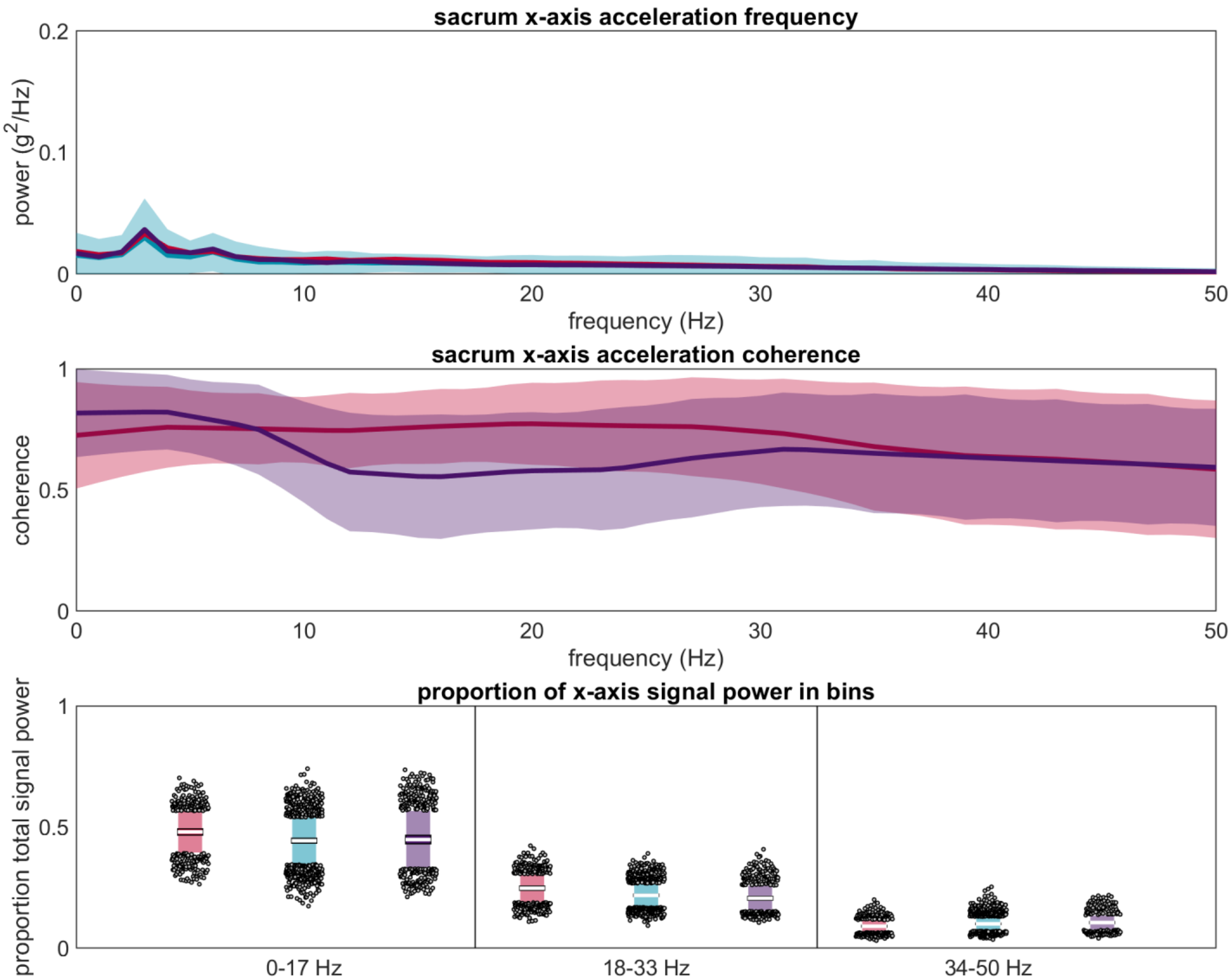

Figure S39: **(Top)** Power spectral density of the sacrum x axis acceleration from 0 to 50 Hz. The blue line represents the mean for the reference IMU, the blue shaded area represents ± 1 SD, and the red and purple lines represent the left-proximal and right-proximal misplaced IMUs. **(Middle)** Magnitude squared coherence between each misplaced IMU and the reference IMU. Plotted as mean ± 1 SD. **(Bottom)** Proportion of total signal power observed in the 0-17 Hz, 18-33 Hz, and 34-50 Hz bins. Central white line represents the mean across trials, dark shaded area represents ± 1.96 SEM (confidence interval; difficult to see due to small size), light shaded area represents ± 1 SD, and grey dots represent trials falling outside ± 1 SD.

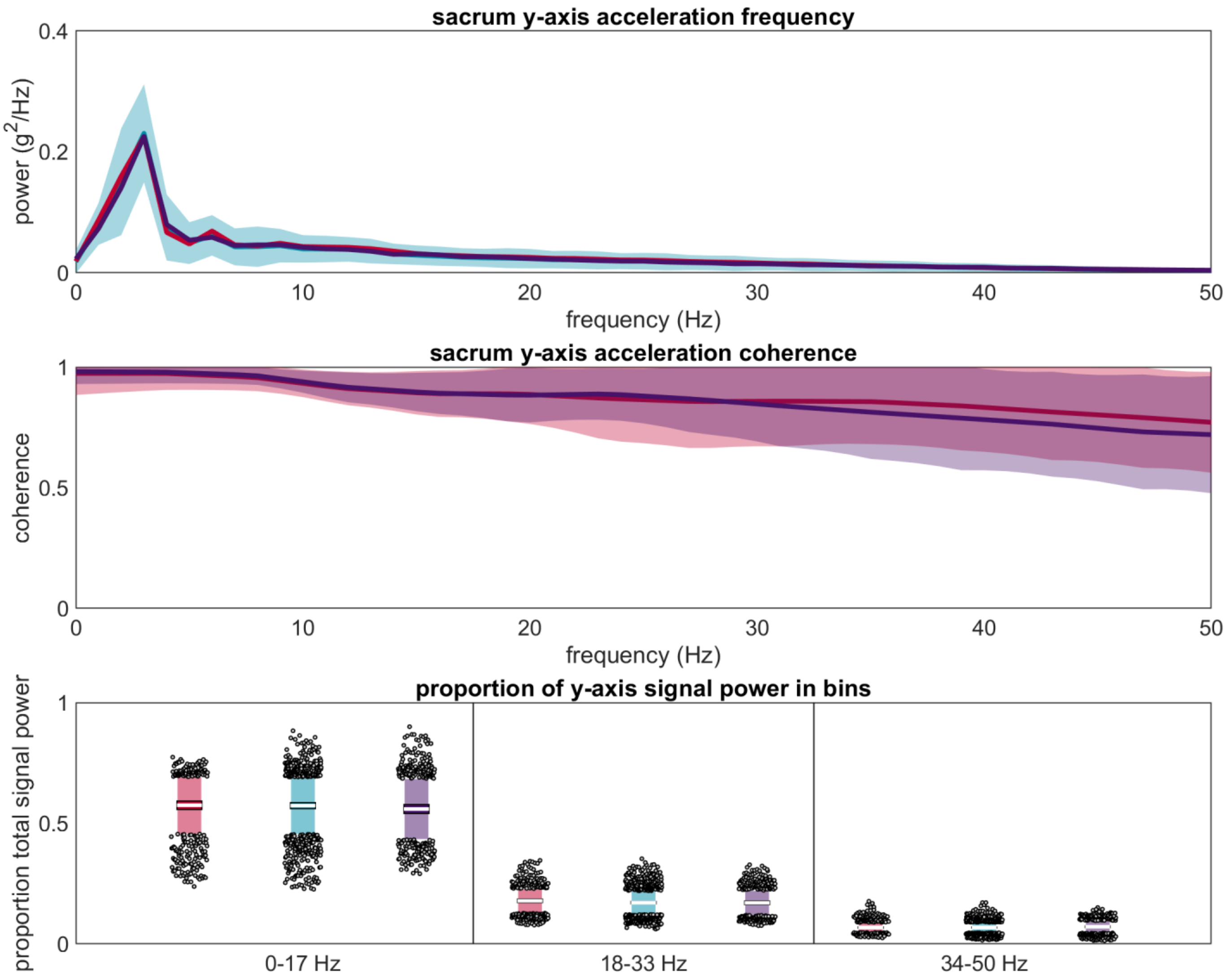

Figure S40: **(Top)** Power spectral density of the sacrum y axis acceleration from 0 to 50 Hz. The blue line represents the mean for the reference IMU, the blue shaded area represents ± 1 SD, and the red and purple lines represent the left-proximal and right-proximal misplaced IMUs. **(Middle)** Magnitude squared coherence between each misplaced IMU and the reference IMU. Plotted as mean ± 1 SD. **(Bottom)** Proportion of total signal power observed in the 0-17 Hz, 18-33 Hz, and 34-50 Hz bins. Central white line represents the mean across trials, dark shaded area represents ± 1.96 SEM (confidence interval; difficult to see due to small size), light shaded area represents ± 1 SD, and grey dots represent trials falling outside ± 1 SD.

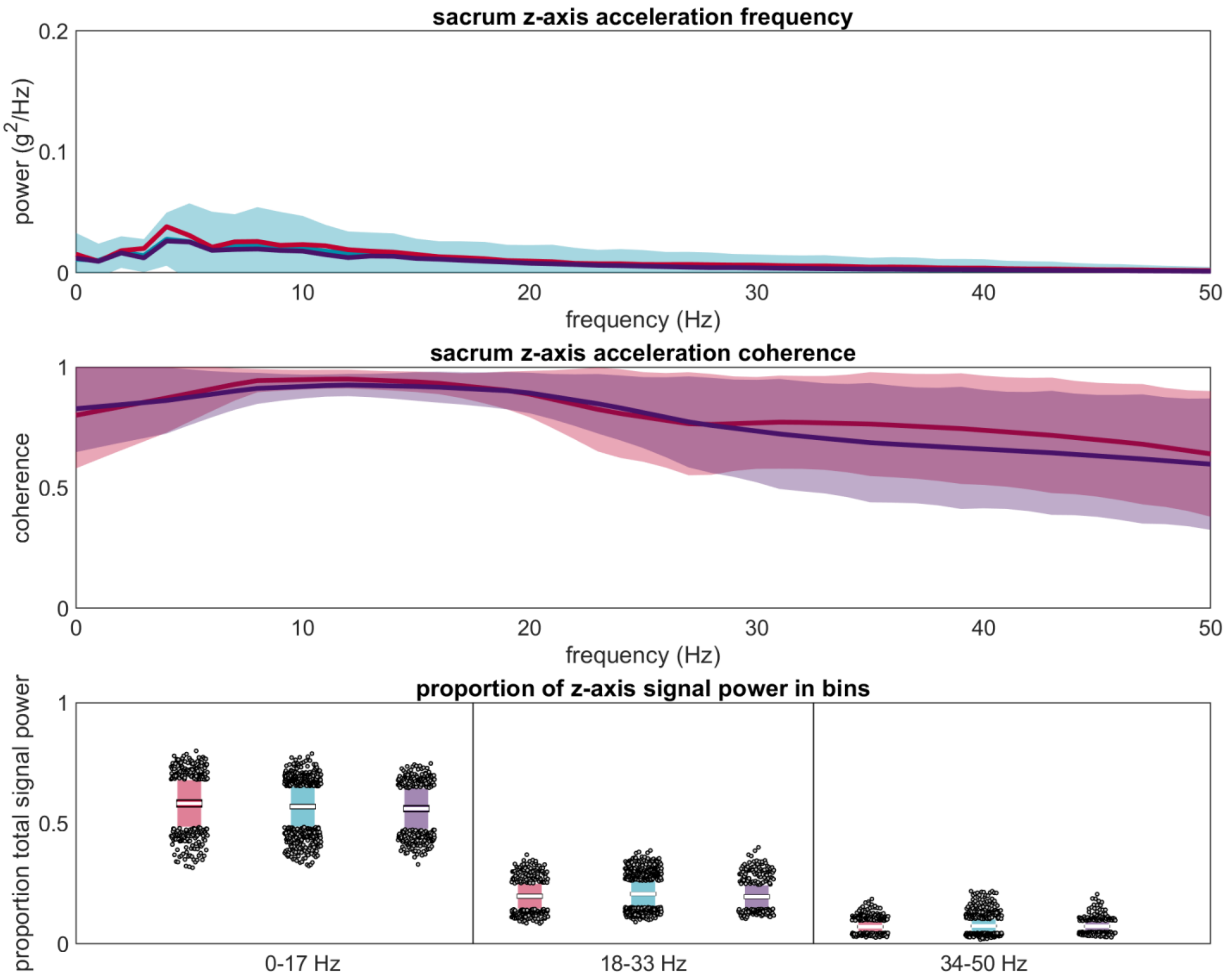

Figure S41: **(Top)** Power spectral density of the sacrum z axis acceleration from 0 to 50 Hz. The blue line represents the mean for the reference IMU, the blue shaded area represents ± 1 SD, and the red and purple lines represent the left-proximal and right-proximal misplaced IMUs. **(Middle)** Magnitude squared coherence between each misplaced IMU and the reference IMU. Plotted as mean ± 1 SD. **(Bottom)** Proportion of total signal power observed in the 0-17 Hz, 18-33 Hz, and 34-50 Hz bins. Central white line represents the mean across trials, dark shaded area represents ± 1.96 SEM (confidence interval; difficult to see due to small size), light shaded area represents ± 1 SD, and grey dots represent trials falling outside ± 1 SD.

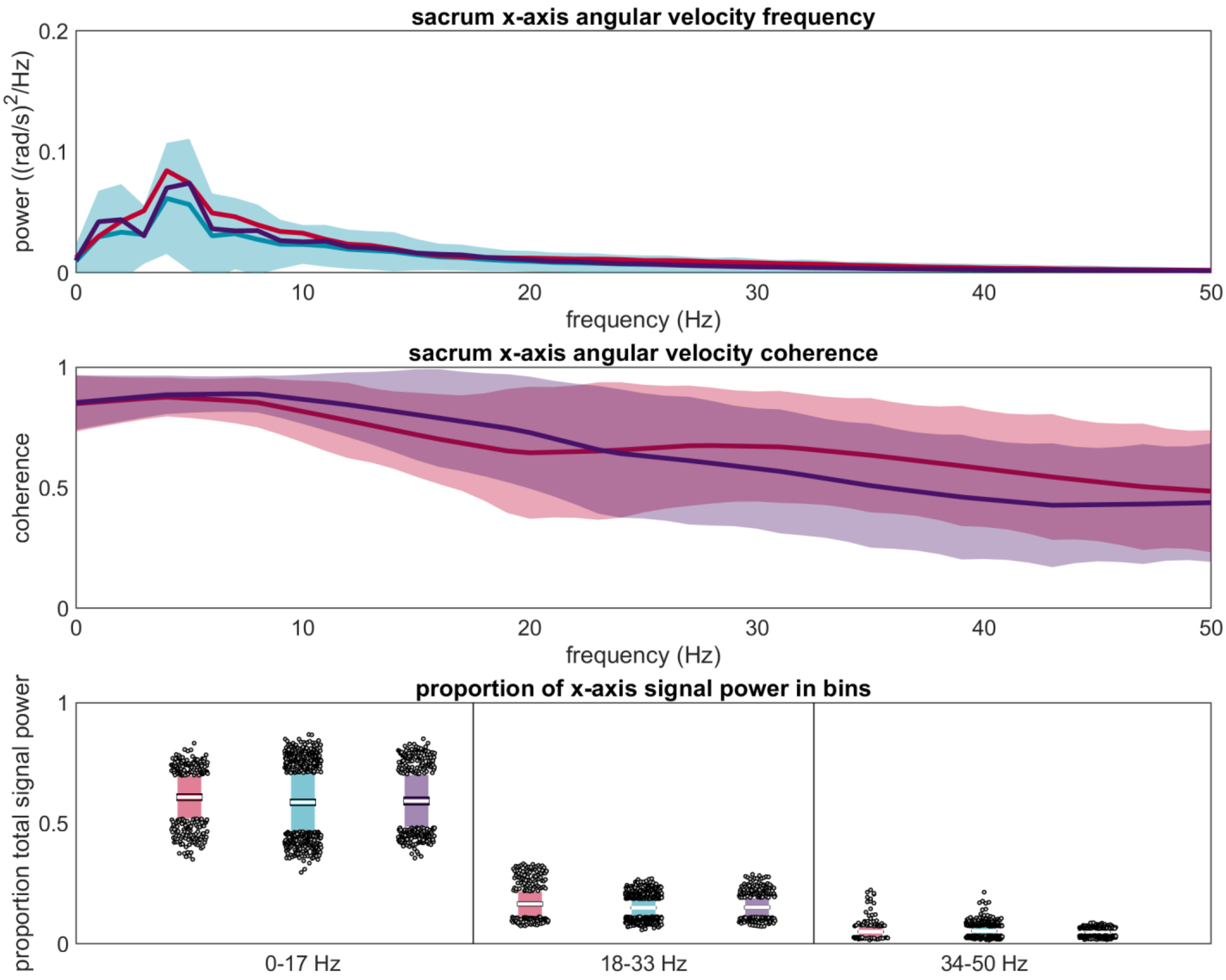

Figure S42: **(Top)** Power spectral density of the sacrum x axis angular velocity from 0 to 50 Hz. The blue line represents the mean for the reference IMU, the blue shaded area represents ± 1 SD, and the red and purple lines represent the left-proximal and right-proximal misplaced IMUs. **(Middle)** Magnitude squared coherence between each misplaced IMU and the reference IMU. Plotted as mean ± 1 SD. **(Bottom)** Proportion of total signal power observed in the 0-17 Hz, 18-33 Hz, and 34-50 Hz bins. Central white line represents the mean across trials, dark shaded area represents ± 1.96 SEM (confidence interval; difficult to see due to small size), light shaded area represents ± 1 SD, and grey dots represent trials falling outside ± 1 SD.

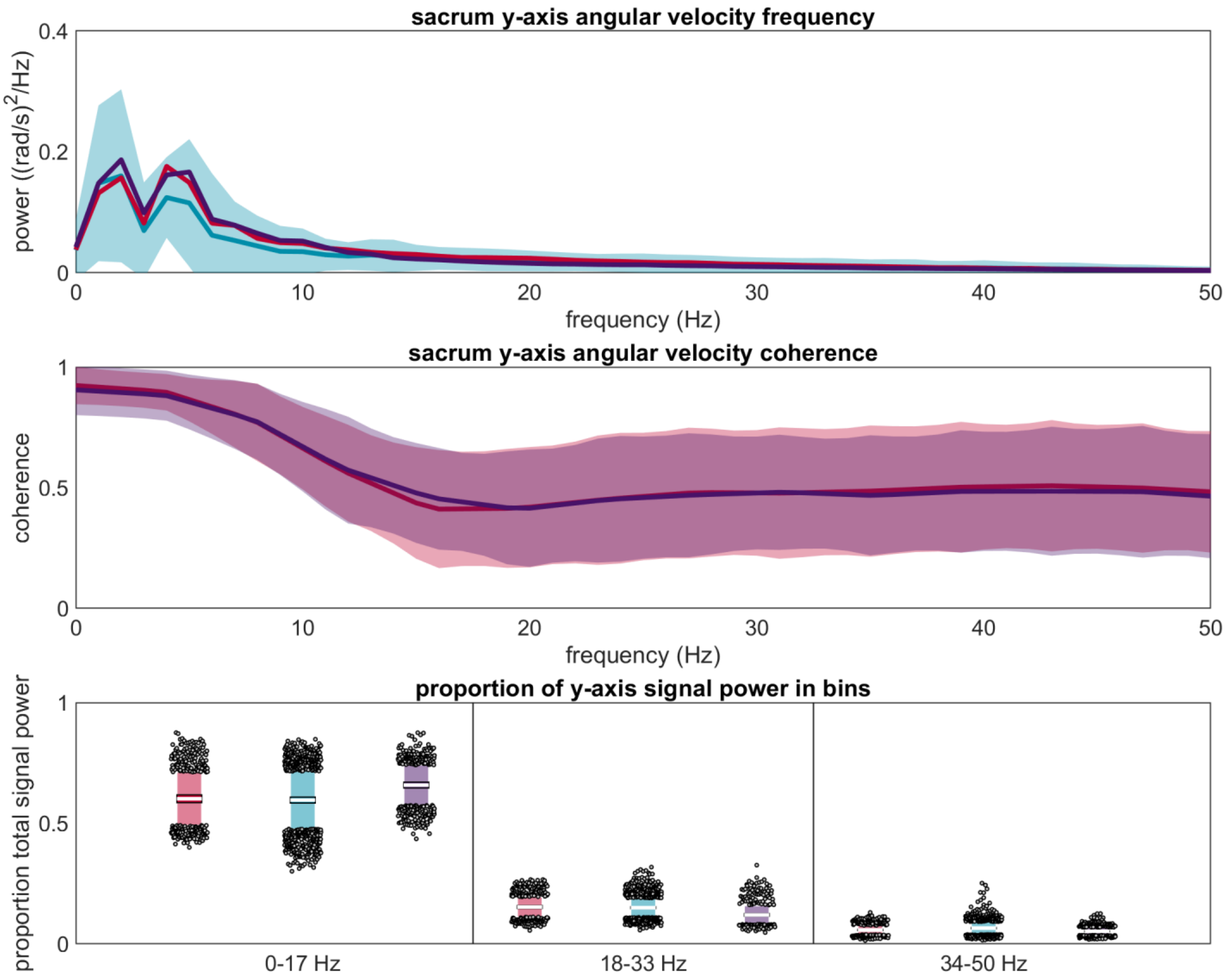

Figure S43: **(Top)** Power spectral density of the sacrum y axis angular velocity from 0 to 50 Hz. The blue line represents the mean for the reference IMU, the blue shaded area represents ± 1 SD, and the red and purple lines represent the left-proximal and right-proximal misplaced IMUs. **(Middle)** Magnitude squared coherence between each misplaced IMU and the reference IMU. Plotted as mean ± 1 SD. **(Bottom)** Proportion of total signal power observed in the 0-17 Hz, 18-33 Hz, and 34-50 Hz bins. Central white line represents the mean across trials, dark shaded area represents ± 1.96 SEM (confidence interval; difficult to see due to small size), light shaded area represents ± 1 SD, and grey dots represent trials falling outside ± 1 SD.

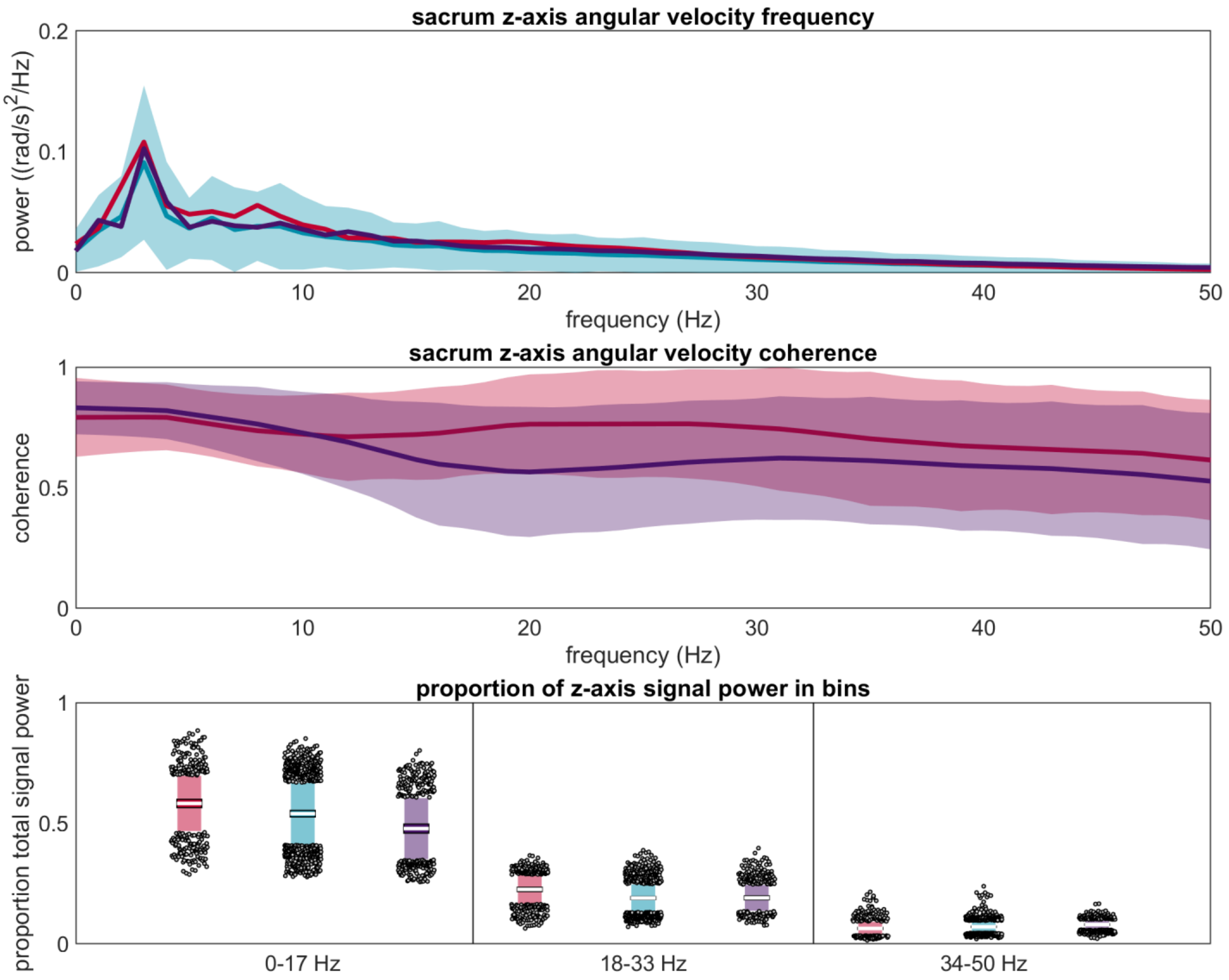

Figure S44: **(Top)** Power spectral density of the sacrum z axis angular velocity from 0 to 50 Hz. The blue line represents the mean for the reference IMU, the blue shaded area represents ± 1 SD, and the red and purple lines represent the left-proximal and right-proximal misplaced IMUs. **(Middle)** Magnitude squared coherence between each misplaced IMU and the reference IMU. Plotted as mean ± 1 SD. **(Bottom)** Proportion of total signal power observed in the 0-17 Hz, 18-33 Hz, and 34-50 Hz bins. Central white line represents the mean across trials, dark shaded area represents ± 1.96 SEM (confidence interval; difficult to see due to small size), light shaded area represents ± 1 SD, and grey dots represent trials falling outside ± 1 SD.

D. Wearable coordinate system results

The analyses presented in the main paper were repeated on the same data expressed in the wearable coordinate system (WCS), as opposed to the segment coordinate system (SCS) (see section A8 for a detailed description of these coordinate systems). An analysis of derived outcome metrics could not be repeated due to difficulties in consistently identifying gait events with the WCS, making a direct comparison between WCS and SCS impossible for those variables. Overall, the analyses revealed that misplacement tended to cause greater differences in the WCS than the SCS, particularly at the shank.

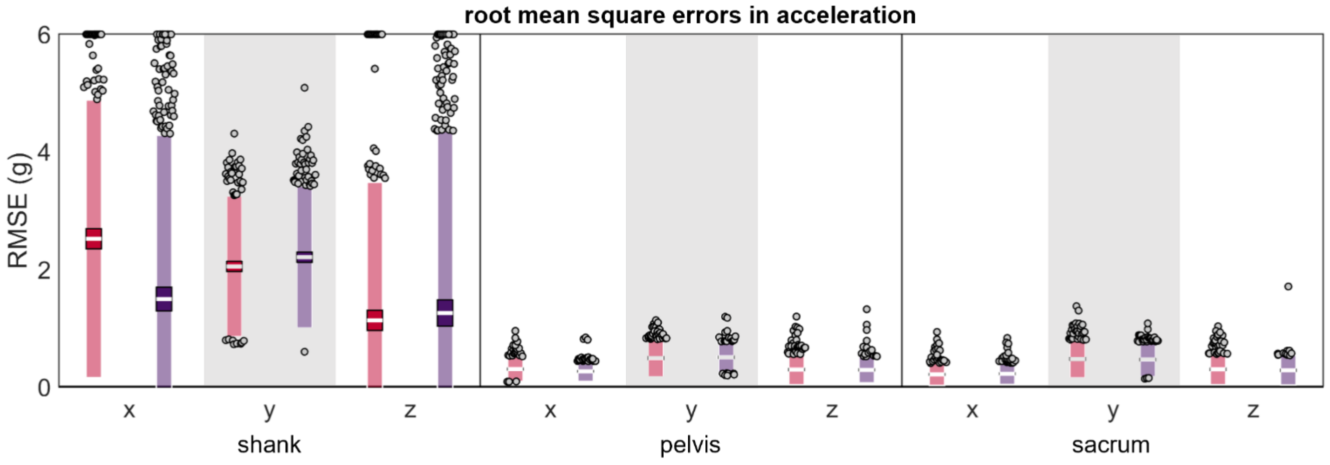

Figure S45: Root mean square error (RMSE) between a measured simultaneously by the reference IMU and the misplaced IMU (red represents anterior- or left-proximal misplacement; purple represents posterior- or right-proximal misplacement). The white line represents the RMSE mean across all trials, the dark-colored box represents the confidence interval about the mean (± 1.96 SEM), the light-colored box represents the limits of agreement (± 1.96 SD), and the grey dots represent trials falling outside the limits of agreement (with trials falling outside the axis limit plotted at the limit).

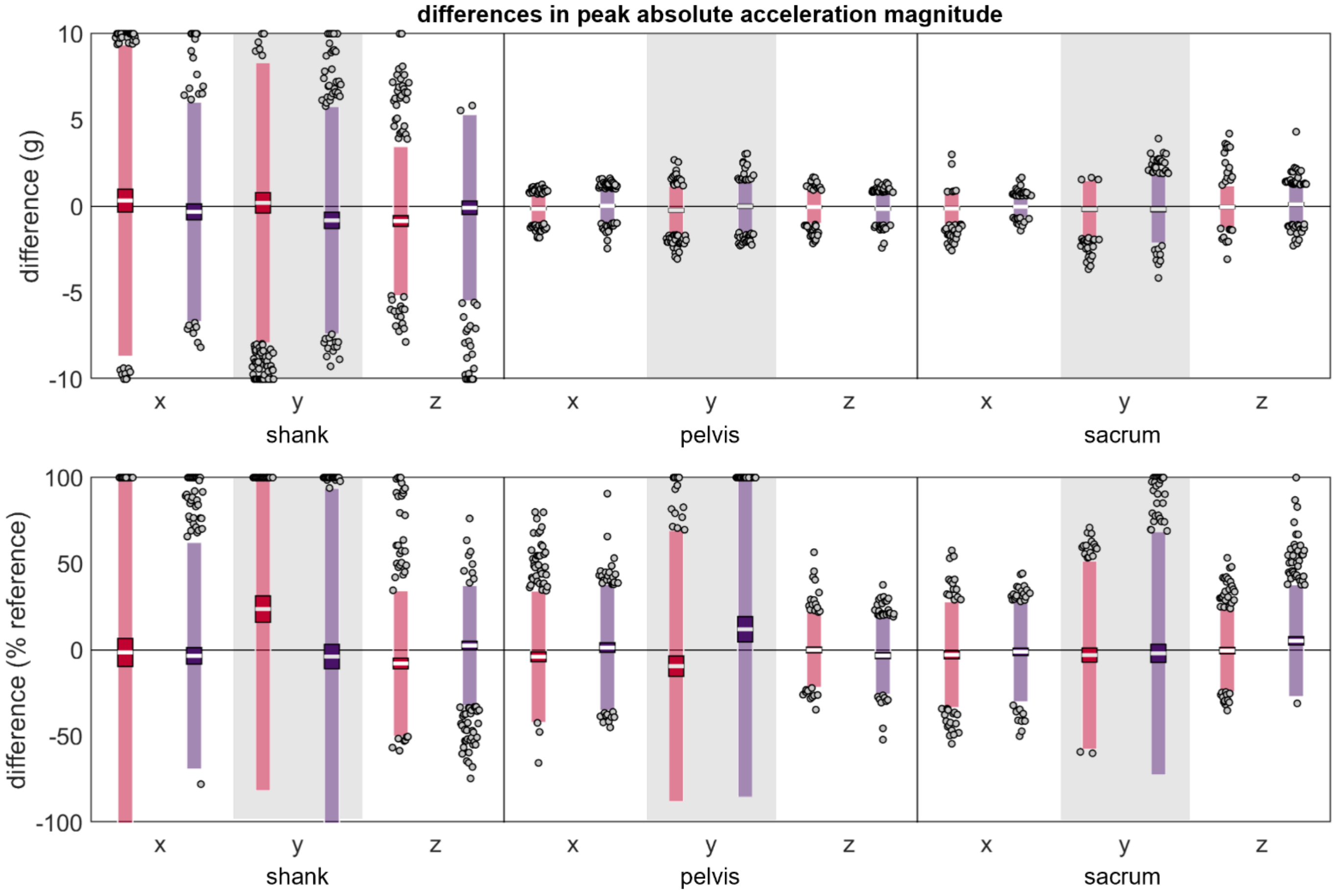

Figure S46: **(A)** absolute and **(B)** normalized differences between peak $|a|$ magnitudes measured simultaneously by the reference IMU and the misplaced IMU (red represents anterior- or left-proximal misplacement; purple represents posterior- or right-proximal misplacement). The white line represents the mean observed difference across trials (bias), the dark-colored box represents the confidence interval about the mean (± 1.96 SEM), the light-colored box represents the limits of agreement (± 1.96 SD), and the grey dots represent trials falling outside the limits of agreement (with trials falling outside the axis limits plotted at the limit). Positive differences indicate the misplaced $|a|$ magnitude was greater than the reference $|a|$ magnitude while negative differences indicate the misplaced $|a|$ magnitude was less than the reference $|a|$ magnitude.

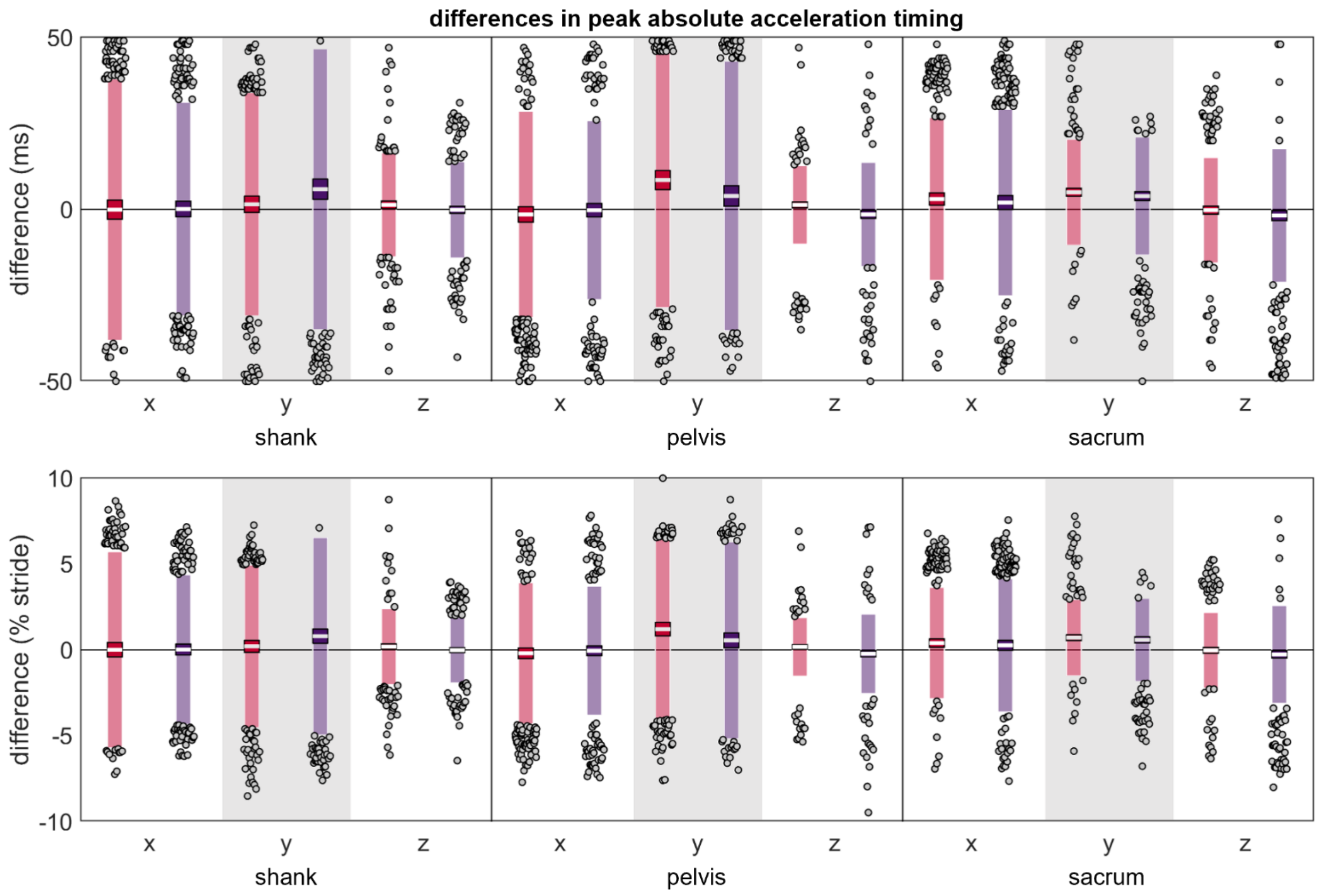

Figure S47: **(A)** absolute and **(B)** normalized differences between peak $|a|$ timings measured simultaneously by the reference IMU and the misplaced IMU (red represents anterior- or left-proximal misplacement; purple represents posterior- or right-proximal misplacement). The white line represents the mean observed difference across trials (bias), the dark-colored box represents the confidence interval about the mean (± 1.96 SEM), the light-colored box represents the limits of agreement (± 1.96 SD), and the grey dots represent trials falling outside the limits of agreement (with trials falling outside the axis limits plotted at the limit). Positive differences indicate the misplaced $|a|$ peak occurring after the reference peak while negative differences indicate the misplaced $|a|$ peak occurring before the reference peak.

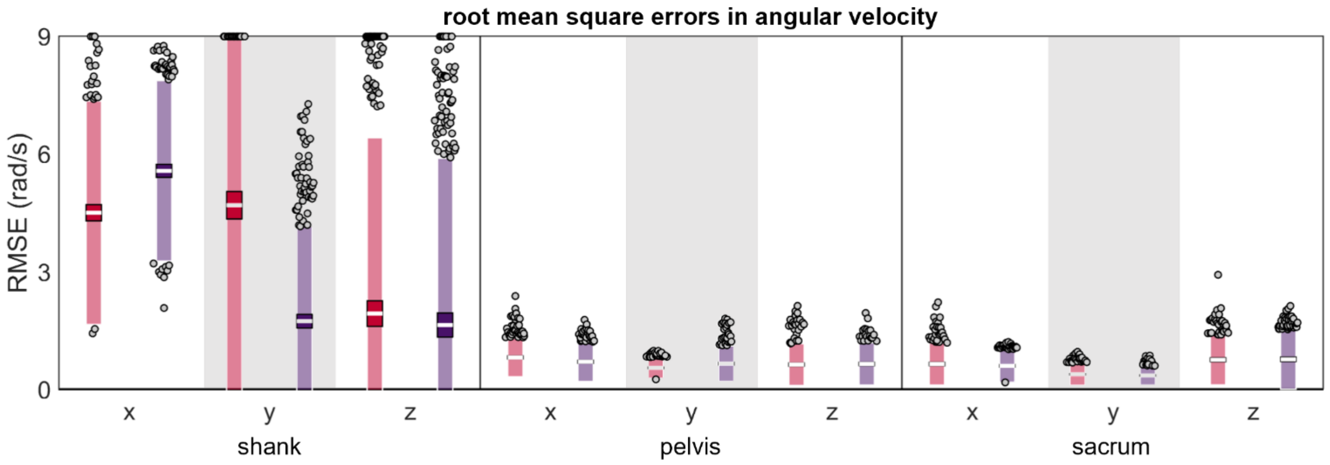

Figure S48: Root mean square error (RMSE) between ω measured simultaneously by the reference IMU and the misplaced IMU (red represents anterior- or left-proximal misplacement; purple represents posterior- or right-proximal misplacement). The white line represents the RMSE mean across all trials, the dark-colored box represents the confidence interval about the mean (± 1.96 SEM), the light-colored box represents the limits of agreement (± 1.96 SD), and the grey dots represent trials falling outside the limits of agreement (with trials falling outside the axis limit plotted at the limit).

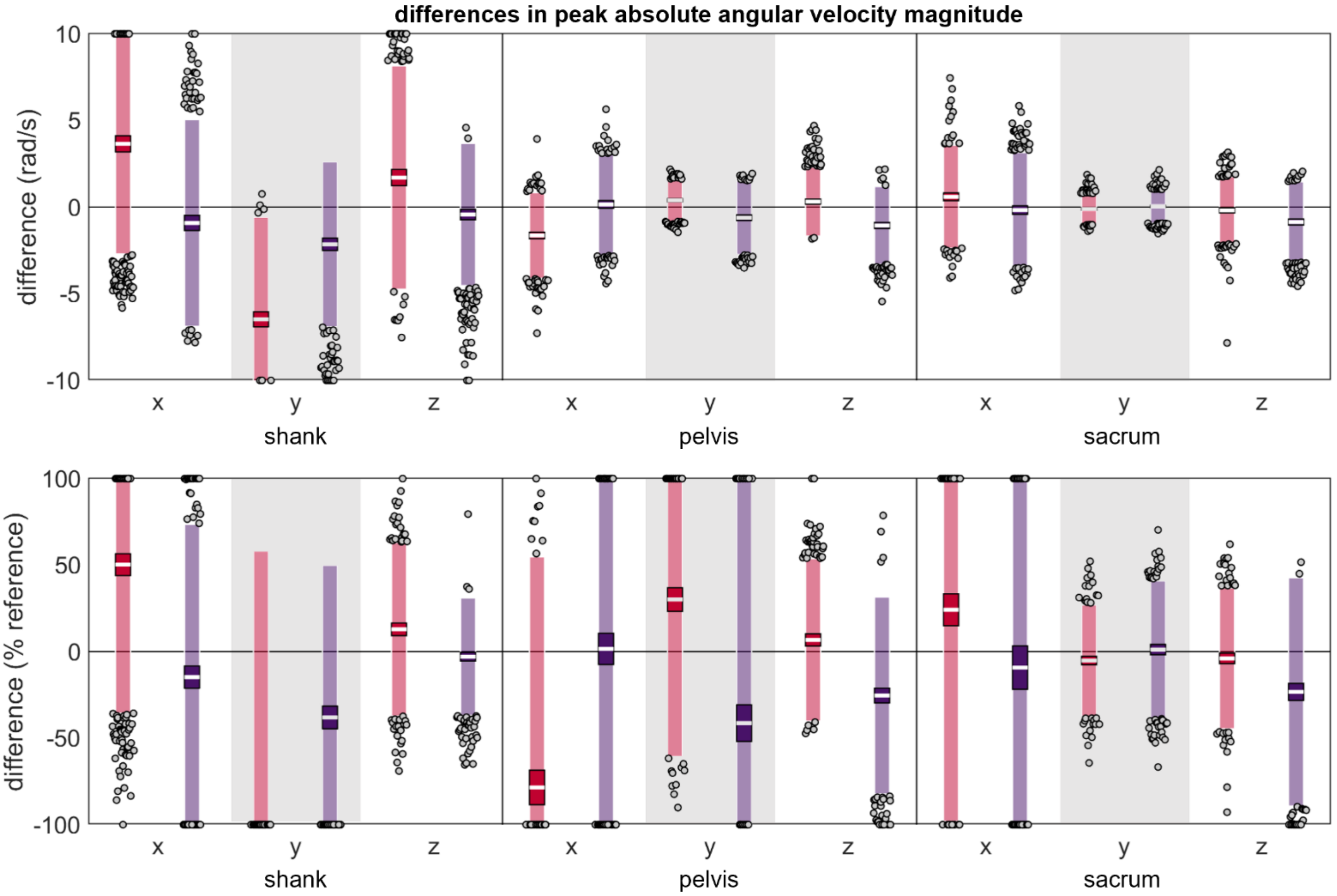

Figure S49: **(A)** absolute and **(B)** normalized differences between peak $|\omega|$ magnitudes measured simultaneously by the reference IMU and the misplaced IMU (red represents anterior- or left-proximal misplacement; purple represents posterior- or right-proximal misplacement). The white line represents the mean observed difference across trials (bias), the dark-colored box represents the confidence interval about the mean (± 1.96 SEM), the light-colored box represents the limits of agreement (± 1.96 SD), and the grey dots represent trials falling outside the limits of agreement (with trials falling outside the axis limits plotted at the limit). Positive differences indicate the misplaced $|\omega|$ magnitude which was greater than the reference $|\omega|$ magnitude, while negative differences indicate the misplaced $|\omega|$ magnitude which was less than the reference $|\omega|$ magnitude.

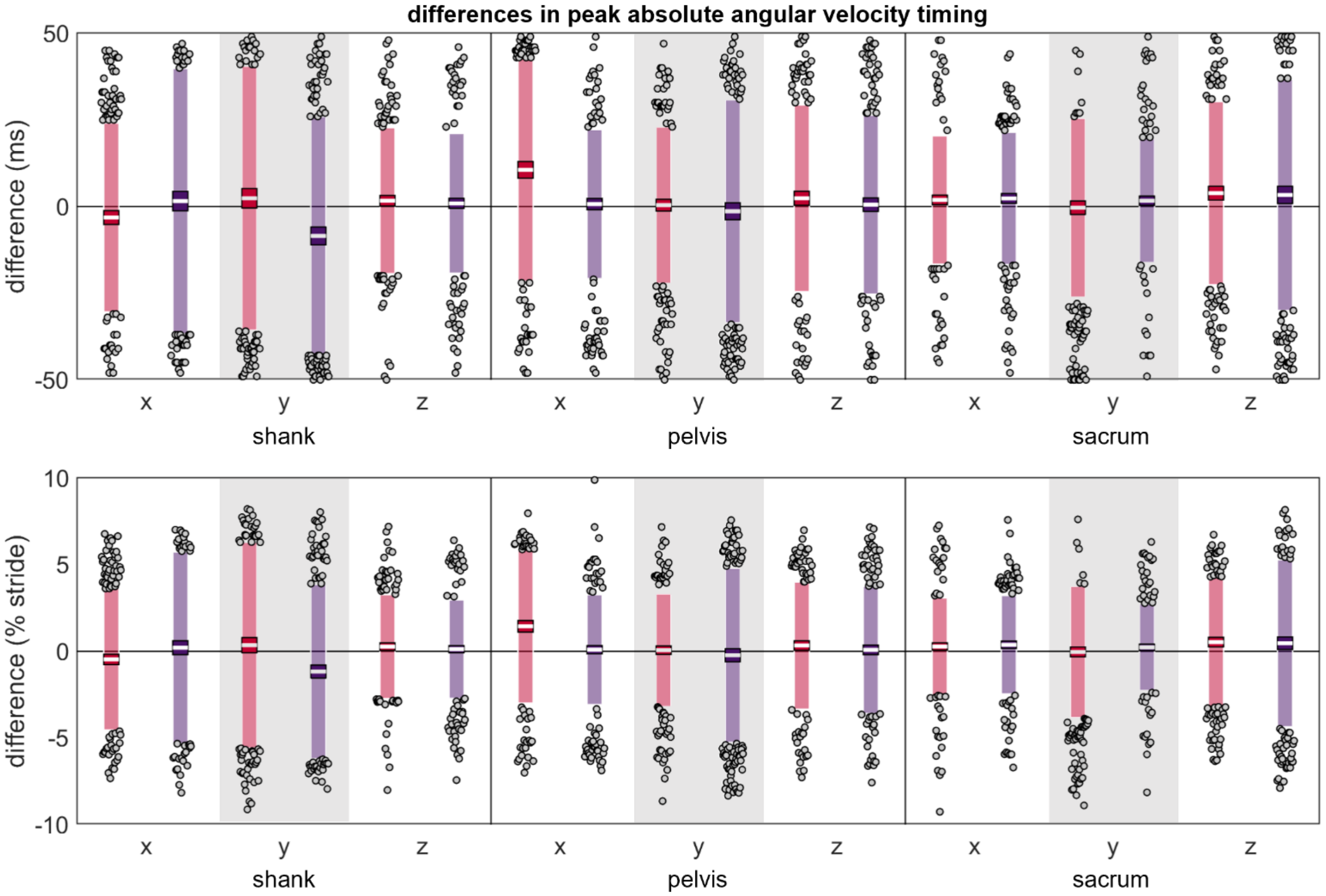

Figure S50: **(A)** absolute and **(B)** normalized differences between peak $|\omega|$ timings measured simultaneously by the reference IMU and the misplaced IMU (red represents anterior- or left-proximal misplacement; purple represents posterior- or right-proximal misplacement). The white line represents the mean observed difference across trials (bias), the dark-colored box represents the confidence interval about the mean (± 1.96 SEM), the light-colored box represents the limits of agreement (± 1.96 SD), and the grey dots represent trials falling outside the limits of agreement (with trials falling outside the axis limits plotted at the limit). Positive differences indicate the misplaced $|\omega|$ peak occurring after the reference peak, while negative differences indicate the misplaced $|\omega|$ peak occurring before the reference peak.

References

[1] United States Geological Survey, "Gravity anamoly map of the continental United States," [Online]. Available: https://mrdata.usgs.gov/gravity/map-us.html#home. [Accessed 5 June 2019].

[2] National Geodtic Survey, "NGS surface gravity prediction," National Oceanic and Atmospheric Administration, [Online]. Available: https://www.ngs.noaa.gov/cgi-bin/grav_pdx.prl. [Accessed 5 June 2019].

[3] C. L. Coolbaugh and D. A. Hawkins, "Standardizing accelerometer-based activity monitor calibration and output reporting," *Journal of Applied Biomechanics,* vol. 30, no. 4, pp. 594-597, 2014.

[4] M. Lafortune, "Three-dimensional acceleration of the tibia during walking and running," *Journal of Biomechanics,* vol. 24, no. 10, pp. 877-886, 1991.

[5] R. Kalman, "A new approach to linear filtering and prediction problems," *Transactions of the ASME–Journal of Basic Engineering,* vol. 82, no. D, pp. 35-45, 1960.

[6] R. Mahony, T. Hamel and J. Pflimlin, "Nonlinear complementary filters on the special orthogonal group," *IEEE Transactions on Automatic Control,* vol. 53, no. 5, pp. 1203-1218, 2008.

[7] S. Madgwick, "An efficient orientation filter for inertial and inertial/magnetic sensor arrays," x-io, Bristol, UK, 2010.

[8] McGinnis and Perkins, "A Highly Miniaturized, Wireless Inertial Measurement Unit for Characterizing the Dynamics of Pitched Baseballs and Softballs," *Sensors,* vol. 12, pp. 11933-11945, 2012.

[9] S. M. Cain, R. S. McGinnis, S. P. Davidson, R. V. Vitalia, N. C. Perkins and S. McLean, "Quantifying performance and effects of load carriage during a challenging balancing task using an array of wireless inertial sensors," *Gait & Posture,* vol. 43, pp. 65-69, 2016.

[10] J. Hammill, T. Derrick and K. Holt, "Shock attenuation and stride frequency during running," *Human Movement Science,* vol. 14, pp. 45-60, 1995.

[11] T. Derrick, J. Hamill and G. Caldwell, "Energy absorption of impacts during running at various stride lengths," *Medicine and Science in Sports and Exercise,* vol. 30, pp. 128-135, 1998.

[12] A. H. Gruber, K. A. Boyer, T. R. Derrick and J. Hamill, "Impact shock frequency components and attenuation in rearfoot and forefoot running," *Journal of Sport and Health Science,* vol. 3, no. 2, pp. 113-121, 2014.

[13] D. Kiernan, R. H. Miller, B. S. Baum, H. J. Kwon and J. K. Shim, "Amputee locomotion: Frequency content of prosthetic vs. intact limb vertical ground reaction forces during running and the effects of filter cut-off frequency," *Journal of Biomechanics,* vol. 60, pp. 248-252, 2017.
